# Review article: A comprehensive review of unusual causes of acute limb compartment syndrome

**DOI:** 10.1111/1742-6723.14098

**Published:** 2022-10-03

**Authors:** William Steadman, Rui Wu, Alistair TM Hamilton, Martin D Richardson, Christopher J Wall

**Affiliations:** ^1^ Orthopaedic Department Toowoomba Hospital Toowoomba Queensland Australia; ^2^ Rural Clinical School The University of Queensland Toowoomba Queensland Australia; ^3^ Emergency Department Toowoomba Hospital Toowoomba Queensland Australia; ^4^ Epworth Clinical School The University of Melbourne Melbourne Victoria Australia

**Keywords:** compartment syndrome, fasciotomy, muscular diseases, trauma

## Abstract

Acute limb compartment syndrome (ALCS) is a surgical emergency that can have serious consequences unless promptly diagnosed and treated, which is particularly challenging when there is an unusual cause. This is a comprehensive review of reported causes of ALCS. From 1068 included articles, we found 299 discrete causes of ALCS including toxins, infections, endocrine pathology, haematological emergencies, malignancy and iatrogenic ALCS. Familiarity with this wide range of ALCS causes may assist in early diagnosis of this limb‐threatening condition.

## Graphical abstract

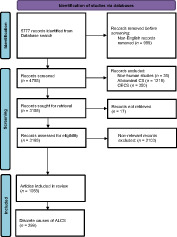

**Key findings**

- ALCS can have a wide range of causes not normally associated with the condition.
- Causes of ALCS include minor trauma, toxins, infections, endocrine pathology, haematological disease, malignancy, and iatrogenesis.
- Awareness of the breadth of ALCS causes, combined with a high index of suspicion, may help facilitate prompt diagnosis of this surgical emergency.

## Introduction

Acute limb compartment syndrome (ALCS) is a surgical emergency that requires prompt recognition and intervention to prevent serious complications. It occurs when there is a pathological increase of intra‐compartmental pressure (ICP), resulting in ischaemia and functional impairment.^1^ ALCS occurs due to either increased compartmental fluid volume or decreased compartmental space. If untreated, a positive feedback loop occurs, with ongoing cell swelling and interstitial oedema resulting in further elevations in ICP.^2^

Prompt diagnosis is critical but often challenging because symptoms vary, and many clinical signs occur late. Emphasis is, therefore, placed on detecting early signs including ischaemic muscle pain, pain on passive movement of distal digits and firm muscle compartments. Conversely, the classical ‘six P's’ of ALCS are late signs that occur in the setting of irreversible muscle damage (the ‘six P's’ are pain, pulselessness, pallor, paraesthesia, paralysis and poikilothermia).^3^

Compartmental pressure measurements can be used when the diagnosis is uncertain.^4^ The role of pressure measurements must be carefully considered, because trauma patients with significant injuries can have raised ICP in the absence of ALCS, and unnecessary fasciotomy may occur when ICP is the only indicator used for diagnosis.^5^, ^6^

Immediate surgical referral and fasciotomy are required as definitive management.^7^ Muscle necrosis can develop within 3 h of injury, and permanent functional deficit rises when fasciotomy is delayed more than 12 h from the onset of symptoms.^8^, ^9^, ^10^, ^11^ Delayed treatment can lead to contracture, fibrosis, stiffness, sensory disturbances, muscle excision, amputation and mortality.^3^

Up to 77% of cases of ALCS are associated with a fracture; however, it also occurs following burns, vascular injury, prolonged immobilisation, infection and haematologic disorders.^12^, ^13^ The aim of this article is to present a comprehensive review of uncommon and rare causes of ALCS, to help with early recognition of this serious condition.

## Methods

A structured Medline search was conducted, from database inception to 21 August 2021. The exploded Medical Subject Heading (MeSH) term ‘compartmental syndromes’ was used, without restrictions. Titles and abstracts were screened for relevance prior to full‐text review. Non‐English, veterinary, abdominal compartment syndrome and chronic exertional compartment syndrome articles were excluded. Where multiple articles for a given cause were found, one article is then cited as a representative report.

## Results

**Figure 1:**
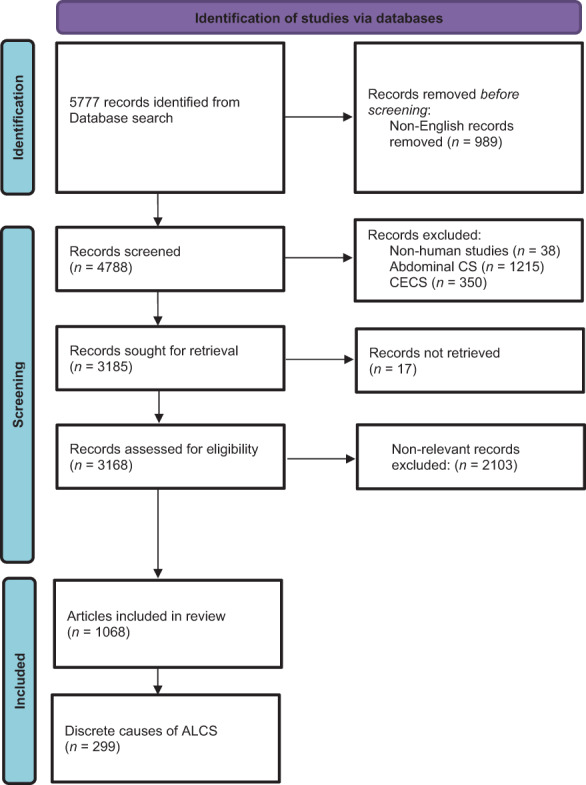
PRISMA flowchart. ALCS, acute limb compartment syndrome; CECS, chronic exertional compartment syndrome (Adapted from Page *et al*.,^14^ with permission).

The literature search found a total of 5777 articles. After excluding 989 non‐English articles, 4788 records were screened. One thousand and sixty‐eight reports of unusual causes of ALCS were identified, with 299 discrete aetiologies. Figure 1 shows the Preferred Reporting Items for Systematic Reviews and Meta‐Analyses (PRISMA) flowchart.^14^

The total number of published articles in each listed category is shown in Table 1. Table S1 details 299 discrete causes and the number of reports with references.

**TABLE 1:** Categorisation of reports of acute limb compartment syndrome (*n* = 1068). Please refer to Table S1 for a detailed list of causes

Category/cause	Reports
Traumatic causes	
Dislocations, muscle tears or sprains	65
Blunt trauma	26
Penetrating trauma	45
Thermal injury	10
Electrical injury	5
Prolonged pressure	65
Toxicological	24
Infective	
Bacterial	51
Viral	19
Bites and stings	40
Medical	
Endocrinological	20
Haematological	48
Genetic conditions	15
Rheumatological	11
Psychiatric	2
Other	17
Obstetric	4
Neonatal	19
Tumour	27
Anatomical	10
Exertional	58
Idiopathic	19
Iatrogenic	
Pressure related	38
Fluid administration	47
Anaesthetic	15
Interventional procedures	78
Orthopaedic procedures	71
Cardiothoracic procedures	16
General surgical procedures	7
Vascular surgical procedures	27
Other surgical procedures	21
Position related	81
Medication	63
Other	4

There were 216 reports of ALCS that were traumatic excluding fractures: 65 related to minor trauma including dislocations, muscle tears or sprains; 26 to blunt trauma; 45 to penetrating trauma; 10 to thermal injuries; five to electrical injuries and 65 to prolonged pressure injuries. Twenty‐four reports were related to toxicological causes. Seventy reports were related to infections and 40 to bite or sting injuries. A total of 113 were medical causes: 20 related to endocrine conditions, 48 to haematological conditions, 15 to genetic conditions, 11 to rheumatological conditions and two to psychiatric conditions. Four reports were related to obstetric causes and there were 19 related to neonatal pathologies. There were 27 tumour‐related reports and 10 related to anatomical variations. Fifty‐eight reports pertained to progression from chronic exertional compartment syndrome and 19 reports were classified as idiopathic by the authors.

There were 468 iatrogenic reports: 38 related to pressure, 47 related to fluid resuscitation and administration and 15 related to anaesthesia. Non‐surgical procedures accounted for 78 reports. Orthopaedic surgical procedures accounted for 71 reports, and non‐orthopaedic surgical procedures had 71 reports. Eighty‐one reports were related to patient positioning during surgery. There were 63 reports related to medication administration.

## Discussion

Our study has identified a wide range of causes of ALCS not typically associated with the condition. In the trauma population, ALCS is usually associated with fracture; however, there were 47 individual reports of ALCS because of muscle tears and 11 cases because of sprain injuries. ALCS also occurred in the setting of penetrating trauma from large animal or human bites. In addition, there were 31 reports of ALCS because of snake bite, along with several cases resulting from wasp stings and insect bites, mostly in children.^15^, ^16^, ^17^

Pro‐ and anti‐coagulant clotting abnormalities are also associated with ALCS, including spontaneous haemorrhage from haemophilia, factor deficiencies and haematological malignancy.^11^, ^18^, ^19^, ^20^, ^21^, ^22^ Platelet deficiency or dysfunction also causes ALCS, and has been reported with human immunodeficiency virus.^23^, ^24^ Extreme thrombocytosis may also cause spontaneous haemorrhage leading to compartment syndrome.^22^, ^25^ Anticoagulant therapy may cause ALCS, especially with minor trauma, such as simple venepuncture or acupuncture.^26^, ^27^, ^28^, ^29^ Hypercoagulable disorders such as protein S deficiency may also present with ALCS related to thrombosis.^18^ Deep vein thrombosis (DVT) accounted for 11 reports, and although predominantly being caused by a provoked DVT in the pre‐treatment phase, ALCS has been reported in unprovoked DVT and with anticoagulation.^30^, ^31^

Type 1 diabetes mellitus was implicated in nine reports of ALCS, without any other clear mechanism. In these cases, patients were diagnosed using a combination of clinical signs, compartment pressures and magnetic resonance imaging, and responded well to fasciotomy. These occurred in both patients with long‐standing and newly diagnosed type 1 diabetes. Some authors suggest an association between diabetes and spontaneous muscle infarction; however, this is difficult to prove.^32^, ^33^ Regardless, an appropriate index of suspicion for ALCS is important in type 1 diabetic patients.

Prolonged limb pressure accounted for several ALCS reports, especially related to drug‐induced altered level of consciousness. Agents involved included alcohol, illicit drugs (opiates, ecstasy, methanol, cannabis, cocaine) and intentional medication overdose. Various illicit drugs may also cause ALCS in the awake patient through local mechanisms including direct cellular toxicity, vasotoxicity or non‐traumatic rhabdomyolysis. Those reported include cathinone ‘bath salts’, benzodiazepines, buprenorphine, barbiturates and heroin.^34^, ^35^, ^36^, ^37^, ^38^ ALCS may occur in patients who have attempted self‐harm or suicide, with reports following deliberate substance ingestion and repeated self‐inflicted soft tissue injury.^39^ Finally, factitious syndrome was reported as a particularly unusual cause of ALCS. In this case, the patient gave a history of subacute minor hand injury inconsistent with their clinical signs of ALCS. Following successful fasciotomy, the patient was found to be self‐mutilating in the hospital, and it was presumed that self‐harm caused compartment syndrome of the hand.^40^

Severe local limb infection, including necrotising fasciitis, may also lead to ALCS in association with infective signs.^41^ Non‐necrotising fasciitis, cellulitis and sepsis were also reported.^42^, ^43^ Group A *Streptococci* and *Staphylococcus aureus* account for most reports, but atypical organisms such as *Vibrio*, *Clostridium* and *Proteus* species are also documented. ALCS due to bacterial pyomyositis from atypical organisms has been reported in immunocompromised oncology patients, including *Aeromonas hydrophila*, *Escherichia coli* and *Klebsiella pneumoniae*, highlighting the importance of sub‐specialty consultation.^44^

Iatrogenic ALCS is reported in a wide variety of medical interventions. Our study found over 150 causes in surgical procedures, most commonly orthopaedic. Extravasation of fluid or medications, including contrast media, accounted for 31 reports. A systematic review of ALCS cases due to extravasation found 76% were either very young or had communication difficulties.^45^ Intraosseous or high volume fluid in resuscitation or burns, or accidental intra‐arterial administration of intravenous drugs may also cause ALCS.^46^, ^47^, ^48^, ^49^, ^50^, ^51^, ^52^, ^53^ Intravenous regional anaesthetic blocks (Bier's blocks) of the upper and lower limb have been reported to cause ALCS, with a systematic review of complications related to this procedure identifying 10 cases in total. Five cases were associated with inadvertent hypertonic saline injection, two with multiple sequential tourniquets, one case with potential excessive tourniquet pressure and one related to an unusually large fracture haematoma.^54^ Non‐invasive blood pressure monitoring has caused ALCS with thrombolytic therapy, prolonged surgical procedures and patients with movement disorders.^55^, ^56^, ^57^, ^58^

Several patient groups are at higher risk of ALCS. A palpable mass along with subtle ALCS symptoms was reported multiple times with limb malignancy, especially sarcoma.^59^, ^60^, ^61^, ^62^ Eleven reports occurred in patients with rheumatological disease, either due to or following elective orthopaedic procedures.^63^, ^64^ ALCS in neonates was reported 19 times due to seven underlying causes. Neonatal patients presented with swelling and loss of function in the affected limb or the sentinel finding of bullous or ulcerated skin lesions.^65^ In this population, the time of onset is often unknown, and therefore there is an association with Volkmann contracture and lifelong impairment of limb function.^65^, ^66^, ^67^

The main strength of our study is the comprehensive literature review, with cases presented by category. The literature search was conducted following PRISMA guidelines to improve validity. We also acknowledge several limitations. Our exclusion criteria may lead to missed reports, most importantly by excluding non‐English language articles. Our study is at risk of publication bias in the primary evidence and report numbers presented should not be interpreted as prevalence or incidence of each cause. We were unable to meet all PRISMA guidelines, as we could not present the study details and risk of bias assessment for each study due to the large number of studies included.

## Conclusion

ALCS is a surgical emergency, which requires emergent fasciotomy to avoid potentially life‐ or limb‐threatening sequelae. Although commonly encountered in the setting of trauma, the present study highlights the fact that ALCS may occur in many clinical scenarios and in settings not normally associated with the condition.

## Supporting information


**Table S1.** Comprehensive list of reported causes of ALCS.

## Data Availability

Data available in article supplementary material.

## References

[1] Matsen FA , Krugmire RB . Compartmental syndromes. Surg. Gynecol. Obstet. 1978; 147: 943–9. 362581

[2] Hargens AR , Mubarak SJ . Current concepts in the pathophysiology, evaluation, and diagnosis of compartment syndrome. Hand Clin. 1998; 14: 371–83. 9742417

[3] Garner MR , Taylor SA , Gausden E , Lyden JP . Compartment syndrome: diagnosis, management, and unique concerns in the twenty‐first century. HSS J. 2014; 10: 143–52. 25050098 10.1007/s11420-014-9386-8 PMC4071472

[4] Wall CJ , Richardson MD , Lowe AJ , Brand C , Lynch J , de Steiger RN . Survey of management of acute, traumatic compartment syndrome of the leg in Australia. ANZ J. Surg. 2007; 77: 733–7. 17685947 10.1111/j.1445-2197.2007.04210.x

[5] Prayson MJ , Chen JL , Hampers D , Vogt M , Fenwick J , Meredick R . Baseline compartment pressure measurements in isolated lower extremity fractures without clinical compartment syndrome. J. Trauma 2006; 60: 1037–40. 16688067 10.1097/01.ta.0000215444.05928.2f

[6] Janzing HM , Broos PL . Routine monitoring of compartment pressure in patients with tibial fractures: beware of overtreatment! Injury 2001; 32: 415–21. 11382429 10.1016/s0020-1383(01)00005-5

[7] Torlincasi AM , Lopez RA , Waseem M . Acute Compartment Syndrome. Treasure Island: StatPearls Publishing LLC, 2021. 28846257

[8] Matsen FA 3rd , Clawson DK . The deep posterior compartmental syndrome of the leg. J. Bone Joint Surg. Am. 1975; 57: 34–9. 1123369

[9] Sheridan GW , Matsen FA 3rd. Fasciotomy in the treatment of the acute compartment syndrome. J. Bone Joint Surg. Am. 1976; 58: 112–5. 1249096

[10] Vaillancourt C , Shrier I , Vandal A *et al*. Acute compartment syndrome: how long before muscle necrosis occurs? CJEM 2004; 6: 147–54. 17433166 10.1017/s1481803500006837

[11] Abdelhalim MA , Shaw CR , Al‐Rub ZA *et al*. Bilateral upper limb compartment syndrome induced by strenuous exercise in a patient with haemophilia A and a low titre inhibitor. Haemophilia 2015; 21: e517–9. 26249323 10.1111/hae.12783

[12] Elliott KG , Johnstone AJ . Diagnosing acute compartment syndrome. J. Bone Joint Surg. 2003; 85: 625–32. 12892179

[13] McQueen MM , Gaston P , Court‐Brown CM . Acute compartment syndrome. J. Bone Joint Surg. 2000; 82‐B: 200–3. 10755426

[14] Page MJ , McKenzie JE , Bossuyt PM *et al*. The PRISMA 2020 statement: an updated guideline for reporting systematic reviews. BMJ 2021; 372: n71. 33782057 10.1136/bmj.n71 PMC8005924

[15] Hardwicke J , Srivastava S . Volkmann's contracture of the forearm owing to an insect bite: a case report and review of the literature. Ann. R. Coll. Surg. Engl. 2013; 95: e36–7. 23484979 10.1308/003588413X13511609955210 PMC4098600

[16] Petratos DV , Galanakos SP , Stavropoulos NA , Anastasopoulos JN . Late compartment syndrome of the hand due to wasp sting in a child. J. Surg. Orthop. Adv. 2011; 20: 202–5. 22214147

[17] Sawyer JR , Kellum EL , Creek AT , Wood GW 3rd. Acute compartment syndrome of the hand after a wasp sting: a case report. J. Pediatr. Orthop. B 2010; 19: 82–5. 19801954 10.1097/BPB.0b013e32832d83f7

[18] Pentz K , Triplet JJ , Johnson DB , Umbel B , Baker TE . Nontraumatic compartment syndrome in a patient with protein S deficiency: a case report. JBJS Case Connect. 2018; 8: e82. 30601765 10.2106/JBJS.CC.18.00055

[19] Dumontier C , Sautet A , Man M , Bennani M , Apoil A . Entrapment and compartment syndromes of the upper limb in haemophilia. J. Hand Surg. Br. 1994; 19: 427–9. 7964091 10.1016/0266-7681(94)90204-6

[20] Jentzsch T , Brand‐Staufer B , Schäfer FP , Wanner GA , Simmen H‐P . Illustrated operative management of spontaneous bleeding and compartment syndrome of the lower extremity in a patient with acquired hemophilia A: a case report. J. Med. Case Rep. 2014; 8: 132. 24886030 10.1186/1752-1947-8-132 PMC4109126

[21] Jones G , Thompson K , Johnson M . Acute compartment syndrome after minor trauma in a patient with undiagnosed mild haemophilia B. Lancet 2013; 382: 1678. 24238556 10.1016/S0140-6736(13)61954-6

[22] Nagase Y , Ueda S , Matsunaga H *et al*. Acute compartment syndrome as the initial manifestation of chronic‐phase chronic myeloid leukemia: a case report and review of the literature. J. Med. Case Rep. 2016; 10: 201. 27443161 10.1186/s13256-016-0985-5 PMC4957314

[23] Desai SS , McCarthy CK , Kestin A , Metzmaker JN . Acute forearm compartment syndrome associated with HIV‐induced thrombocytopenia. J. Hand Surg. 1993; 18: 865–7. 10.1016/0363-5023(93)90055-8 8228059

[24] Navaneethan U , Kemmer N , Neff GW . Acquired platelet dysfunction with spontaneous intramuscular hematoma and compartment syndrome in cirrhosis. Minerva Gastroenterol. Dietol. 2008; 54: 445–9. 19047984

[25] Teng CJ , Huang CT , Huang YC , Hong YC , Wang WS , Tzeng CH . Too many platelets to cause compartment syndrome. QJM 2011; 104: 993–4. 20923789 10.1093/qjmed/hcq186

[26] Nixon RG , Brindley GW . Hemophilia presenting as compartment syndrome in the arm following venipuncture. A case report and review of the literature. Clin. Orthop. Relat. Res. 1989; (244): 176–81. 2663286

[27] Roberge RJ , McLane M . Compartment syndrome after simple venipuncture in an anticoagulated patient. J. Emerg. Med. 1999; 17: 647–9. 10431955 10.1016/s0736-4679(99)00059-1

[28] Smith DL , Walczyk MH , Campbell S . Acupuncture‐needle‐induced compartment syndrome. West. J. Med. 1986; 144: 478–9. 3716412 PMC1306683

[29] Shah N , Hing C , Tucker K , Crawford R . Infected compartment syndrome after acupuncture. Acupunct. Med. 2002; 20: 105–6. 12216597 10.1136/aim.20.2-3.105

[30] Newman PA , Deo S . Non‐traumatic compartment syndrome secondary to deep vein thrombosis and anticoagulation. BMJ Case Rep. 2014; 2014: bcr2013201689. 10.1136/bcr-2013-201689 PMC3902344 24443334

[31] Lamborn DR , Schranz C . Compartment syndrome as a complication of ileofemoral deep venous thrombosis: a case presentation. Am. J. Emerg. Med. 2014; 32: 192.e1–2. 10.1016/j.ajem.2013.08.050 24091199

[32] Woolley SL , Smith DRK . Acute compartment syndrome secondary to diabetic muscle infarction: case report and literature review. Eur. J. Emerg. Med. 2006; 13: 113–6. 16525245 10.1097/01.mej.0000192048.04729.45

[33] Chautems RC , Irmay F , Magnin M , Morel P , Hoffmeyer P . Spontaneous anterior and lateral tibial compartment syndrome in a type I diabetic patient: case report. J. Trauma 1997; 43: 140–1. 9253926 10.1097/00005373-199707000-00034

[34] Saleh A , Tittley J , Anand S . Limb‐threatening ischemia in a young man with cathinone “Bath Salt” intoxication: a case report. Ann. Vasc. Surg. 2016; 36: 294.e1–5. 10.1016/j.avsg.2016.03.025 27423726

[35] Levine M , Levitan R , Skolnik A . Compartment syndrome after “bath salts” use: a case series. Ann. Emerg. Med. 2013; 61: 480–3. 23318022 10.1016/j.annemergmed.2012.11.021

[36] Rutgers PH , van der Harst E , Koumans RK . Surgical implications of drug‐induced rhabdomyolysis. Br. J. Surg. 1991; 78: 490–2. 2032112 10.1002/bjs.1800780432

[37] Partanen TA , Vikatmaa P , Tukiainen E , Lepäntalo M , Vuola J . Outcome after injections of crushed tablets in intravenous drug abusers in the Helsinki University Central Hospital. Eur. J. Vasc. Endovasc. Surg. 2009; 37: 704–11. 19328024 10.1016/j.ejvs.2009.01.016

[38] Franc‐Law JM , Rossignol M , Vernec A , Somogyi D , Shrier I . Poisoning‐induced acute atraumatic compartment syndrome. Am. J. Emerg. Med. 2000; 18: 616–21. 10999581 10.1053/ajem.2000.9271

[39] Eskander MB , MacDonald R . Acute tibial compartment syndrome secondary to psychosomatic disorder. J. R. Army Med. Corps 1994; 140: 97–8. 8907840 10.1136/jramc-140-02-11

[40] Bachy M , Moncany AH , Tournier C *et al*. Factitious disorders in the hand‐Main diagnostic traps highlighted with 3 cases. Hand Surg. Rehabil. 2018; 37: 110–3. 29292110 10.1016/j.hansur.2017.10.237

[41] Kleshinski J , Bittar S , Wahlquist M , Ebraheim N , Duggan JM . Review of compartment syndrome due to group A streptococcal infection. Am. J. Med. Sci. 2008; 336: 265–9. 18794622 10.1097/MAJ.0b013e318165650a

[42] Wong K , Nicholson DJ , Gray R . Lower limb compartment syndrome arising from fulminant Streptococcal sepsis. ANZ J. Surg. 2005; 75: 728–9. 16076343 10.1111/j.1445-2197.2005.03507.x

[43] Toney J , Donovan S , Adelman V , Adelman R . Non‐necrotizing streptococcal cellulitis as a cause of acute, atraumatic compartment syndrome of the foot: a case report. J. Foot Ankle Surg. 2016; 55: 418–22. 25981442 10.1053/j.jfas.2015.02.010

[44] Cone LA , Lamb RB , Graff‐Radford A *et al*. Pyomyositis of the anterior tibial compartment. Clin. Infect. Dis. 1997; 25: 146–8. 9243048 10.1086/514497

[45] Pare JR , Moore CL . Intravenous infiltration resulting in compartment syndrome: a systematic review. J. Patient Saf. 2018; 14: e6–8. 26241617 10.1097/PTS.0000000000000233

[46] Galpin RD , Kronick JB , Willis RB , Frewen TC . Bilateral lower extremity compartment syndromes secondary to intraosseous fluid resuscitation. J. Pediatr. Orthop. 1991; 11: 773–6. 1960204 10.1097/01241398-199111000-00014

[47] Block EF , Dobo S , Kirton OC . Compartment syndrome in the critically injured following massive resuscitation: case reports. J. Trauma 1995; 39: 787–91. 7473979 10.1097/00005373-199510000-00036

[48] Dulhunty JM , Boots RJ , Rudd MJ , Muller MJ , Lipman J . Increased fluid resuscitation can lead to adverse outcomes in major‐burn injured patients, but low mortality is achievable. Burns 2008; 34: 1090–7. 18468802 10.1016/j.burns.2008.01.011

[49] Davies SJ , Wall CJ , Winter MW , Landy M . A limb threatening complication of intravenous fluid resuscitation. ANZ J. Surg. 2013; 83: 493–4. 23735137 10.1111/ans.12204

[50] Schanzer H , Gribetz I , Jacobson JH 2nd. Accidental intra‐arterial injection of penicillin G. A preventable catastrophe. JAMA 1979; 242: 1289–90. 480544

[51] Robinson P , Leow JM , Brown I . Compartment syndrome of the hand following iatrogenic intra‐arterial administration of epinephrine during cardiopulmonary resuscitation. BMJ Case Rep. 2021; 14: e241320. 10.1136/bcr-2020-241320 PMC7986765 33753391

[52] Funk L , Grover D , de Silva H . Compartment syndrome of the hand following intra‐arterial injection of heroin. J. Hand Surg. Br. 1999; 24: 366–7. 10433458 10.1054/jhsb.1997.0218

[53] Lindfors NC , Vilpponen L , Raatikainen T . Complications in the upper extremity following intra‐arterial drug abuse. J. Hand Surg. Eur. Vol. 2010; 35: 499–504. 20237182 10.1177/1753193408094438

[54] Guay J . Adverse events associated with intravenous regional anesthesia (Bier block): a systematic review of complications. J. Clin. Anesth. 2009; 21: 585–94. 20122591 10.1016/j.jclinane.2009.01.015

[55] Sutin KM , Longaker MT , Wahlander S , Kasabian AK , Capan LM . Acute biceps compartment syndrome associated with the use of a noninvasive blood pressure monitor. Anesth. Analg. 1996; 83: 1345–6. 8942614 10.1097/00000539-199612000-00040

[56] Vidal P , Sykes PJ , O'Shaughnessy M , Craddock K . Compartment syndrome after use of an automatic arterial pressure monitoring device. Br. J. Anaesth. 1993; 71: 902–4. 8280563 10.1093/bja/71.6.902

[57] Alford JW , Palumbo MA , Barnum MJ . Compartment syndrome of the arm: a complication of noninvasive blood pressure monitoring during thrombolytic therapy for myocardial infarction. J. Clin. Monit. Comput. 2002; 17: 163–6. 12455731 10.1023/a:1020736206507

[58] Celoria G , Dawson JA , Teres D . Compartment syndrome in a patient monitored with an automated blood pressure cuff. J. Clin. Monit. 1987; 3: 139–41. 3585434 10.1007/BF00858363

[59] Goldfarb CA , Jaffe KA , Chivers FS , Listinsky CM . Compartment syndrome. An unusual etiology. Clin. Orthop. Relat. Res. 1998; 356: 248–53. 9917691

[60] Scheipl S , Leithner A , Radl R *et al*. Myeloid sarcoma presenting in muscle‐tissue of the lower limb: unusual origin of a compartment‐syndrome. Am. J. Clin. Oncol. 2007; 30: 658–9. 18091063 10.1097/01.coc.0000189704.33839.f9

[61] Shapiro SA , Brindley GW . Synovial sarcoma presenting as an acute compartment syndrome. J. Bone Joint Surg. Am. 1995; 77: 1249–50. 7642673 10.2106/00004623-199508000-00017

[62] Ali SH , Chughtai H , Alali F , Diaczok B , Verardi M . Wrist drop: an atypical presentation of renal cell carcinoma. Am. J. Med. Sci. 2011; 342: 170–3. 21795958 10.1097/MAJ.0b013e31821d4544

[63] Jo D , Pompa T , Khalil A , Kong F , Wetz R , Goldstein M . Rheumatoid myositis leading to acute lower extremity compartment syndrome: a case‐based review. Clin. Rheumatol. 2015; 34: 1813–6. 24810700 10.1007/s10067-014-2657-4

[64] Noorpuri BS , Shahane SA , Getty CJ . Acute compartment syndrome following revisional arthroplasty of the forefoot: the dangers of ankle‐block. Foot Ankle Int. 2000; 21: 680–2. 10966367 10.1177/107110070002100809

[65] Ragland R 3rd , Moukoko D , Ezaki M , Carter PR , Mills J . Forearm compartment syndrome in the newborn: report of 24 cases. J. Hand Surg. 2005; 30: 997–1003. 10.1016/j.jhsa.2005.06.003 16182057

[66] Tetreault AK , Axibal DP , Scott FA . Neonatal compartment syndrome treated within the first 24 hours of life. Orthopedics 2018; 41: e731–3. 29913028 10.3928/01477447-20180613-06

[67] Plancq MC , Buisson P , Deroussen F , Krim G , Collet LM , Gouron R . Successful early surgical treatment in neonatal compartment syndrome: case report. J. Hand Surg. 2013; 38: 1185–8. 10.1016/j.jhsa.2013.03.029 23664365

