# Supplementary material for: Review article: A comprehensive review of unusual causes of acute limb compartment syndrome
Source: Emerg Med Australas. 2022 Oct 3;34(6):871–6. doi: 10.1111/1742-6723.14098 (PMC9828535; doi:10.1111/1742-6723.14098)
Supplement: Supplementary file 1 — Table S1. Comprehensive list of reported causes of ALCS. [file EMM-34-871-s001.docx]

Supplementary Table S1: Reported causes of ALCS with references

| **Category/Cause** | **Reports** |
| --- | --- |
| **TRAUMATIC CAUSES** |  |
| - Dislocations | 7 |
| - Elbow^68^ |  |
| - Knee ^69^ |  |
| - Hip ^70^ |  |
| - Shoulder^71^ |  |
| - Muscle tears | 47 |
| - Peroneus Longus ^72^ |  |
| - Gastrocnemius ^73^ |  |
| - Tibialis Anterior ^74^ |  |
| - Triceps ^75^ |  |
| - Hamstring ^76^ |  |
| - Quadriceps ^77^ |  |
| - Flexor digitorum profundus ^78^ |  |
| - Flexor digitorum superficialis ^79^ |  |
| - Extensor digitorum communis ^80^ |  |
| - Biceps Brachii ^81^ |  |
| - Brachioradialis ^82^ |  |
| - Sprains | 11 |
| - Lateral Ankle ^83^ |  |
| - Medial Ankle ^84^ |  |
| - Blunt injury | 26 |
| - Thigh ^85^ |  |
| - Forearm ^86^ |  |
| - Gluteal ^87^ |  |
| - Leg ^88^ |  |
| - Penetrating injury^89-91^ | 4 |
| - Gunshot injury^92^ | 8 |
| - Traction injury^93-95^ | 13 |
| - Crush injury^96^ | 11 |
| - Vacuum injury^97^ | 5 |
| Vascular injury^98^ | 3 |
| - Inferior gluteal artery ^99^ |  |
| - Superior gluteal artery ^100^ |  |
| - Blast injury ^101^ | 1 |
|  |  |
| **Thermal Injury** |  |
| - Burns^102^ | 6 |
| - Heat stroke^103^ | 2 |
| - Frost bite^104^ | 2 |
|  |  |
| **Electrical Injury** |  |
| - Electrocution^105^ | 4 |
| - Lightning strike^106^ | 1 |
|  |  |
| **Prolonged Pressure** |  |
| - Accidental^107^ | 11 |
| - Alcoholic coma^108^ | 11 |
| - Medication overdose ^109^ | 11 |
| - Codeine ^110^ |  |
| - Barbiturates ^111^ |  |
| - Benzodiazepines ^36^ |  |
| - Illicit drug induced coma | 31 |
| - Heroin + various opiates ^37, 112^ |  |
| - Ecstasy ^113^ |  |
| - Methanol ^114^ |  |
| - Cannabis ^115^ |  |
| - Cocaine ^116^ |  |
| - Occupational ^117^ | 1 |
|  |  |
| **TOXICOLOGICAL** |  |
| - Accidental | 5 |
| - Carbon monoxide^118^ |  |
| - Drug overdose | 7 |
| - Ergotamine ^119^ |  |
| - Salicylate ^120^ |  |
| - Theophylline ^121^ |  |
| - Diphenhydramine ^122^ |  |
| - Oral hypoglycaemics ^123^ |  |
| - Self-harm | 7 |
| - Hydrocarbon injection ^124^ |  |
| - Injected bleach, rubbing alcohol, lighter fluid ^38^ |  |
| - Brodifacoum ingestion ^125^ |  |
| - Insecticide injection ^126^ |  |
| - Intra-arterial drug injection | 3 |
| - Temazepam ^127^ |  |
| - Midazolam ^53^ |  |
| - Amiodarone ^128^ |  |
| - Adrenaline (epinephrine) ^51^ |  |
| - Bath Salts^34^ | 2 |
|  |  |
| **INFECTIVE** |  |
| **Bacterial** | 51 |
| - Staphylococcus aureus pyomyositis ^129^ |  |
| - - Methicillin-resistant staphylococcus aureus pyomyositis ^130^ |  |
| - - Methicillin-sensitive staphylococcus aureus osteomyelitis ^131^ |  |
| - Streptococcus pyogenes pyomyositis ^132^ |  |
| - - Group A Streptococcus (GAS) fasciitis ^133^ |  |
| - - GAS sepsis ^42^ |  |
| - - GAS cellulitis ^134^ |  |
| - Aeromonas Hydrophilia ^44^ |  |
| - Escherichia coli ^44^ |  |
| - Klebsiella pneumoniae ^44^ |  |
| - Salmonella enteritis ^135^ |  |
| - Vibrio cholerae ^136^ |  |
| - Vibrio vulnificus ^137^ |  |
| - Neisseria Meningitidis ^138^ |  |
| - Pseudomonas aeruginosa ^139^ |  |
| - Moraxella lacunata ^140^ |  |
| - Anthrax ^141^ |  |
| - Plesiomonas shigelloides ^142^ |  |
| - Proteus mirabillis ^143^ |  |
| - Clostridium septicum^144^ |  |
| - Purpura fulminans related to various organisms ^145^ |  |
| - Tuberculous pyomyositis^146^ |  |
|  |  |
| **Viral** | 19 |
| - Human Immunodeficiency Virus (HIV) ^147^ |  |
| - Parainfluenza ^148^ |  |
| - Influenza A ^149^ |  |
| - Varicella ^150, 151^ ^152^ |  |
| - Influenza B ^153^ |  |
| - Coxsackie B ^154^ |  |
| - Crimean-Congo haemorrhagic fever virus ^155^ |  |
| - Dengue Fever ^156^ |  |
| - COVID-19 ^157^ |  |
|  |  |
| **BITES** |  |
| - Human^158^ | 1 |
| - Animal | 33 |
| - Dog ^159^ |  |
| - Stingray^160^ |  |
| - Snake^161^ |  |
| - Insect | 6 |
| - Wasp Sting^16^ |  |
| - Black Widow Spider ^162^ |  |
|  |  |
| **MEDICAL** |  |
| **Endocrinological** |  |
| - Diabetes | 10 |
| - Type 1 Diabetes Mellitus ^33^ |  |
| - Diabetes Insipidus ^163^ |  |
| - - Hypothyroidism^164^ | 7 |
| - Thyroid hormone withdrawal ^165^ |  |
| - Electrolyte disturbance | 3 |
| - Hyponatremia secondary to polydipsia ^166^ |  |
| - Hypocalcaemic tetany ^167^ |  |
|  |  |
| **Haematological** |  |
| - Hereditary Angioedema ^168^ | 2 |
| - - Clotting disorders | 24 |
| - Haemophilia A ^169^ |  |
| - Haemophilia B ^21^ |  |
| - Factor VII deficiency ^170^ |  |
| - Factor XIII deficiency ^171^ |  |
| - Protein S deficiency ^18^ |  |
| - - Coagulopathic states | 4 |
| - Diffuse idiopathic coagulation ^172^ |  |
| - HIV induced thrombocytopenia ^23^ |  |
| - Essential Thrombocythemia ^25^ |  |
| - End-stage liver disease ^173^ |  |
| - - Sickle cell trait/disease^174^ | 7 |
| - - Deep Vein Thrombosis^175^ | 11 |
| - Phlegmasia cerulea dolens ^176^ |  |
|  |  |
| **Genetic conditions** |  |
| - McCardle’s Disease^177^ | 5 |
| - Neurofibromatosis 1^178^ | 3 |
| - GYG1 mutation^179^ | 1 |
| - Duchenne’s Muscular Dystrophy ^180^ | 1 |
| - Ehlers-Danlos syndrome^181^ | 5 |
|  |  |
| **Rheumatological** | 11 |
| - Granulomatosis with polyangiitis^182^ |  |
| - Henoch Schonlein purpura.^183^ |  |
| - Systemic lupus erythematosus-induced vasculitis ^184^ |  |
| - HIV vasculitis ^184^ |  |
| - Buerger Disease ^185^ |  |
| - Polyarteritis nodosa ^186^ |  |
| - Rheumatoid myositis^63^ |  |
| - Systemic sclerosis^187^ |  |
| - RS3PE ^188^ |  |
| - Eosinophilic myositis ^189^, eosinophilic fasciitis ^190^ |  |
|  |  |
| **Psychiatric** | 2 |
| - Dermatitis artefacta ^39^ |  |
| - Factitious disorder ^40^ |  |
|  |  |
| **Other** |  |
| - Ergotamine ^191^ | 1 |
| - Vasomotor instability ^192^ | 1 |
| - Systemic capillary leak syndrome ^193^ | 12 |
| - Familial rhabdomyolysis^194^ | 3 |
|  |  |
| **OBSTETRIC** | 4 |
| - Uncomplicated labour ^195^ |  |
| - Post-partum haemorrhage ^196^ |  |
| - Post-caesarean section ^197^ |  |
|  |  |
| **NEONATAL** | 19 |
| - Neonatal compartment syndrome ^198^ |  |
| - Harlequin ichthyosis ^199^ |  |
| - Entrapment in uterine diverticulum ^200^ |  |
| - Spontaneous axillary artery thrombosis ^201^ |  |
| - Acute haemorrhagic oedema of infancy ^202^ |  |
| - Disseminated intravascular coagulation ^203^ |  |
| - Neonatal sepsis and scleremia neonatorum^204^ |  |
|  |  |
| **TUMOUR** |  |
| - Benign | 3 |
| - Hemangioma^205^ |  |
| - Hamartoma ^206^ |  |
| - Malignant | 4 |
| - Large cell lymphoma of skeletal muscle^207^ |  |
| - Myeloid sarcoma ^60^ |  |
| - Synovial Sarcoma ^61^ |  |
| - Metastatic | 2 |
| - Renal cell carcinoma^62^ |  |
| - Malignant melanoma ^208^ |  |
| - Infiltration | 8 |
| - Chronic myeloid leukaemia ^209^ |  |
| - Non-Hodgkin’s lymphoma ^210^ |  |
| - Leukaemic infiltration ^211, 212^ |  |
| - Plasmacytoma ^213^ |  |
| - Other | 10 |
| - Osteocartilaginous exostosis ^214^ |  |
| - Ganglion cyst ^215^ |  |
| - Calcific tendinitis ^216^ |  |
| - Ruptured Baker’s cyst ^217^ |  |
|  |  |
| **ANATOMICAL** |  |
| - Arterial aneurysm/pseudoaneurysm injury | 8 |
| - Popliteal aneurysm^218^, popliteal cyst ^219^ |  |
| - Tibial artery aneurysm ^186^ |  |
| - Profunda femoris artery pseudoaneurysm ^220^ |  |
| - Radial artery pseudoaneurysm ^221^ |  |
| - Ulnar artery mycotic aneurysm ^222^ |  |
| - Brachial artery pseudoaneurysm ^223^ |  |
| - Tibiofibular synostosis^224^ | 1 |
| - Abnormal muscular anatomy^225^ | 1 |
|  |  |
| **EXERTIONAL** | 58 |
| - Forearm^226^ |  |
| - Gluteal^227^ |  |
| - Thigh^228^ |  |
| - Leg^229^ |  |
| - Foot^230^ |  |
|  |  |
| **IDIOPATHIC** | 19 |
| - Foot ^231^ |  |
| - Leg ^232^ |  |
| - Forearm ^233^ |  |
| - Isolated peroneal compartment ^234^ |  |
| - Thenar eminence ^235^ |  |
|  |  |
| **IATROGENIC** |  |
| **Pressure Related** |  |
| - Plaster^236^ | 5 |
| - Bandage^237^ | 3 |
| - Brace^238^ | 1 |
| - Skin traction^239^ | 3 |
| - Tourniquet^240^ | 6 |
| - Compression stockings^241^ | 1 |
| - Elastic wraps over compression stockings^242^ | 1 |
| - Sequential compression device^243^ | 2 |
| - NIBP monitoring^57^ | 4 |
| - Pneumatic antishock garments^244^ | 12 |
|  |  |
| **Fluid Resuscitation** |  |
| - Extravasation of intravenous fluid^245^ | 31 |
| - Calcium gluconate extravasation ^246^ |  |
| - Sodium valproate ^247^ |  |
| - Mannitol ^248^ |  |
| - Noradrenaline extravasation ^249^ |  |
| - Propofol extravasation ^250^ |  |
| - Phenytoin bolus^251^ |  |
| - Intraosseous infusion^252^ | 13 |
| - Massive fluid resuscitation^47^ | 2 |
| - Fluid resuscitation in burns^48^ | 1 |
|  |  |
| **Anaesthetic** |  |
| - Intravenous regional anaesthesia^253^ | 5 |
| - Haematoma block for distal radius fracture^254^ | 1 |
| - Peripheral nerve block^255^ | 2 |
| - Epidural anaesthesia with motor blockade^256^ | 1 |
| - Malignant hyperthermia^257^ | 6 |
|  |  |
| **Interventional** |  |
| - Arterial puncture/cannulation/catheterisation^258-273^ | 27 |
| - Venous puncture/cannulation/catheterisation^26, 27, 273-278^ | 8 |
| - Arterio-venous (AV) fistula access^279^ | 6 |
| - Inferior vena cava filter^280, 281^ | 2 |
| - Arterial embolisation^282^ | 1 |
| - Cardiopulmonary bypass^283, 284^ | 5 |
| - Intra-aortic balloon pumping^285^ | 5 |
| - Hauser procedure^286-288^ | 3 |
| - Fogarty catheter^289^ | 1 |
| - Magnetic Resonance Imaging^290^ | 1 |
| - Contrast extravasation ^165^ | 6 |
| - Sclerotherapy^291, 292^ | 2 |
| - Cutaneous laser surgery^293^ | 1 |
| - Electromyography^294-296^ | 3 |
| - Bone marrow biopsy^297^ | 3 |
| - Punch biopsy^298^ | 1 |
| - Extra-corporeal membrane oxygenation^284^ | 3 |
|  |  |
| **Orthopaedic Procedures** | 71 |
| - Distal radius osteotomy^299^ |  |
| - Distal ulnar osteotomy^300^ |  |
| - Wrist arthrodesis^301^ |  |
| - Full-thickness skin graft harvest from forearm for Dupuytren’s surgery ^302^ |  |
| - Anterior & posterior lumbar spine fusion^303^ |  |
| - Lumbar disc replacement^304^ |  |
| - Resection of pelvic tumour^305^ |  |
| - Total hip arthroplasty ^306^ |  |
| - Revision total hip arthroplasty ^307^ |  |
| - Hip resurfacing^306^ |  |
| - Total knee arthroplasty^308^ |  |
| - Uni-compartmental knee arthroplasty^309^ |  |
| - Femoral head core decompression ^310^ |  |
| - Knee arthroscopy^311^ |  |
| - Posterior cruciate ligament reconstruction^312^ |  |
| - Distal femoral osteotomy^313^ |  |
| - High tibial osteotomy^314^ |  |
| - Patellar tendon transplantation^315^ |  |
| - Removal of tibial osteochondroma^316^ |  |
| - Tibialis anterior hernia repair^317^ |  |
| - Achilles tendon repair^318^ |  |
| - Plantaris tendon harvest^319^ |  |
| - Ankle arthroscopy^320^ |  |
| - Revision forefoot arthroplasty^64^ |  |
| - Intramedullary tibial pulse lavage for osteomyelitis^321^ |  |
| - Wound irrigation^322^ |  |
| - Skeletal traction^323^ |  |
| - Motor evoked potential monitoring during spinal surgery^324^ |  |
| - Hydrocortisone injection for plantar fasciitis ^325^ |  |
|  |  |
| **Cardiothoracic Procedures** | 16 |
| - Coronary artery bypass graft harvest^326^ |  |
| - Cardiac valve replacement^327^ |  |
| - Simultaneous cardiac & renal transplant^328^ |  |
| - Excision of lung lesion^329^ |  |
|  |  |
| **General Surgical Procedures** | 7 |
| - Post-low anterior resection ^330^ |  |
| - Excision of retroperitoneal tumour^331^ |  |
| - Laparoscopic surgery^332, 333^ |  |
| - Common iliac & popliteal artery thrombosis post-open nephrouretectomy ^334^ |  |
|  |  |
| **Vascular Surgical Procedures** | 27 |
| - Major vascular surgery^335-342^ |  |
| - Revascularisation injury^343-348^ |  |
| - Creation of AV fistula^349, 350^ |  |
|  |  |
| **Other Surgical Procedures** |  |
| - Maxillofacial surgery^351-353^ | 3 |
| - Caesarean section^354, 355^ | 2 |
| - Trans obturator tape procedure^356^ | 1 |
| - Free flap surgery | 12 |
| - Anterolateral thigh flap^357^ |  |
| - Free fibula osteocutaneous flap ^358^ |  |
| - Latissimus dorsi flap compartment syndrome ^359^ |  |
| - Calf implants^360^ | 1 |
| - Unknown aetiology^361, 362^ | 2 |
|  |  |
| **Position Related** |  |
| - Lithotomy ^363^ | 54 |
| - Lloyd-Davies position^364^ | 3 |
| - Hemilithotomy^365^ | 6 |
| - Knee chest position^366^ | 5 |
| - Lateral decubitus^367^ | 2 |
| - Prone ^368^ | 2 |
| - Supine position^369^ | 5 |
| - Arms tucked tightly beside body^370^ | 1 |
| - Robotic urological surgery^371^ | 3 |
|  |  |
| **Medication** | 63 |
| - Anticoagulation |  |
| - Warfarin ^372^ |  |
| - Dual anti-platelet therapy ^373^ |  |
| - Plavix ^374^ |  |
| - Enoxaparin ^375^ |  |
| - Dalteparin ^376^ ^377^ |  |
| - Fondaparinux ^378^ |  |
| - Thrombolysis^379^ |  |
| - Isolated limb perfusion^380^ |  |
| - Iloprost infusion^381^ |  |
| - Intra-muscular injection |  |
| - Bicillin^382^ |  |
| - B12 injection ^383^ |  |
| - Accidental intra-arterial injection of penicillin ^50^ |  |
| - Non-steroidal anti-inflammatory^384^ |  |
| - Cyclosporin^385^ |  |
| - Cholesterol lowering agents |  |
| - Gemfibrozil ^386^ |  |
| - Simvastatin ^387^ |  |
| - Neuroleptics^388^, Serotonin Syndrome^389^ |  |
| - Lithium^390^ |  |
|  |  |
| **Other** |  |
| - Continuous passive motion^391^ | 1 |
| - Acupuncture^392^ | 3 |

**References for Supplementary Table S1**

[68] Queipo-de-Llano Temboury A, Lara JM, Fernadez-de-Rota A, Queipo-de-Llano E. Anterior elbow dislocation with potential compartment syndrome: a case report. *Techniques In Hand & Upper Extremity Surgery*. 2007; **11**: 18-23.

[69] Steele HL, Singh A. Vascular injury after occult knee dislocation presenting as compartment syndrome. *The Journal Of Emergency Medicine*. 2012; **42**: 271-74.

[70] Taylor BC, Dimitris C, Tancevski A, Tran JL. Gluteal compartment syndrome and superior gluteal artery injury as a result of simple hip dislocation: a case report. *The Iowa Orthopaedic Journal*. 2011; **31**: 181-86.

[71] Yen CY, Yeh WL, Tu YK. Inferior dislocation of the glenohumeral joint combined with the compartment syndrome of the upper arm: case report. *Changgeng Yi Xue Za Zhi / Changgeng Ji Nian Yi Yuan = Chang Gung Medical Journal / Chang Gung Memorial Hospital*. 1998; **21**: 358-61.

[72] Gwynne Jones DP, Theis JC. Acute compartment syndrome due to closed muscle rupture. *The Australian And New Zealand Journal Of Surgery*. 1997; **67**: 227-28.

[73] Anouchi YS, Parker RD, Seitz WH, Jr. Posterior compartment syndrome of the calf resulting from misdiagnosis of a rupture of the medial head of the gastrocnemius. *The Journal Of Trauma*. 1987; **27**: 678-80.

[74] Machani B, Narayan B, Casserly HB. Closed avulsion of the tibialis anterior: an unusual cause of compartment syndrome. *Injury*. 2000; **31**: 738-39.

[75] Brumback RJ. Compartment syndrome complicating avulsion of the origin of the triceps muscle. A case report. *The Journal Of Bone And Joint Surgery. American Volume*. 1987; **69**: 1445-47.

[76] Kwong Y, Patel J, Ramanathan EBS. Spontaneous complete hamstring avulsion causing posterior thigh compartment syndrome. *British Journal Of Sports Medicine*. 2006; **40**: 723-24.

[77] Burns BJ, Sproule J, Smyth H. Acute compartment syndrome of the anterior thigh following quadriceps strain in a footballer. *British Journal Of Sports Medicine*. 2004; **38**: 218-20.

[78] Choi G, Huang JL, Fowble V, Tucci J. Volar forearm compartment syndrome following flexor digitorum profundus muscle rupture in a 3-year-old girl. *American Journal Of Orthopedics (Belle Mead, N.J.)*. 2008; **37**: E108-E09.

[79] Gainor BJ. Closed avulsion of the flexor digitorum superficialis origin causing compartment syndrome. A case report. *The Journal Of Bone And Joint Surgery. American Volume*. 1984; **66**: 467-67.

[80] Crawford B, Comstock S. Acute compartment syndrome of the dorsal forearm following noncontact injury. *CJEM*. 2010; **12**: 453-56.

[81] Fung DA, Frey S, Grossman RB. Rare case of upper arm compartment syndrome following biceps tendon rupture. *Orthopedics*. 2008; **31**: 494-94.

[82] Nakamura G, Abe M, Kumano H. Acute Compartment Syndrome of the Forearm Secondary to Hematoma after Playing Golf. *The journal of hand surgery Asian-Pacific volume*. 2019; **24**: 93-95.

[83] Cheng LY, Niedfeldt MW, Lachacz J, Raasch WG. Acute, isolated lateral compartment syndrome after ankle inversion injury. *Clinical Journal Of Sport Medicine: Official Journal Of The Canadian Academy Of Sport Medicine*. 2007; **17**: 151-52.

[84] Cortina J, Amat C, Selga J, Corona PS. Isolated medial foot compartment syndrome after ankle sprain. *Foot and ankle surgery : official journal of the European Society of Foot and Ankle Surgeons*. 2014; **20**: e1-e2.

[85] Gorman PW, McAndrew MP. Acute anterior compartmental syndrome of the thigh following contusion. A case report and review of the literature. *Journal Of Orthopaedic Trauma*. 1987; **1**: 68-70.

[86] Anderson WJ, Sterling DA. Posttraumatic compartment syndrome of the dorsal forearm: an unusual case. *Orthopedics*. 1997; **20**: 265-66.

[87] Brumback RJ. Traumatic rupture of the superior gluteal artery, without fracture of the pelvis, causing compartment syndrome of the buttock. A case report. *The Journal Of Bone And Joint Surgery. American Volume*. 1990; **72**: 134-37.

[88] Kemp MA, Barnes JR, Thorpe PL, Williams JL. Avulsion of the perforating branch of the peroneal artery secondary to an ankle sprain: a cause of acute compartment syndrome in the leg. *The Journal Of Foot And Ankle Surgery: Official Publication Of The American College Of Foot And Ankle Surgeons*. 2011; **50**: 102-03.

[89] Gillooly JJ, Hacker A, Patel V. Compartment syndrome as a complication of a stab wound to the thigh: a case report and review of the literature. *Emergency Medicine Journal: EMJ*. 2007; **24**: 780-81.

[90] Morin RJ, Swan KG, Tan V. Acute forearm compartment syndrome secondary to local arterial injury after penetrating trauma. *The Journal Of Trauma*. 2009; **66**: 989-93.

[91] Schnall SB, Holtom PD, Silva E. Compartment syndrome associated with infection of the upper extremity. *Clinical Orthopaedics And Related Research*. 1994: 128-31.

[92] Moed BR, Fakhouri AJ. Compartment syndrome after low-velocity gunshot wounds to the forearm. *Journal Of Orthopaedic Trauma*. 1991; **5**: 134-37.

[93] Docker C, Titley OG. A case of forearm compartment syndrome following a ring avulsion injury. *Injury*. 2002; **33**: 274-75.

[94] Soong M, DaSilva M. Acute forearm compartment syndrome secondary to digit avulsion injury. A case report. *The Journal Of Bone And Joint Surgery. American Volume*. 2009; **91**: 435-37.

[95] Pantle HA, Chanmugam A. Case report: acute forearm compartment syndrome due to sudden, forceful supination of the hand. *The Journal Of Emergency Medicine*. 2010; **39**: e143-e46.

[96] Dahlin LB, Ljungberg E, Esserlind A-L. Injuries of the hand and forearm in young children caused by steam roller presses in laundries. *Scandinavian Journal Of Plastic And Reconstructive Surgery And Hand Surgery / Nordisk Plastikkirurgisk Forening [And] Nordisk Klubb For Handkirurgi*. 2008; **42**: 43-47.

[97] Bluman EM, Tashjian RZ, Graves PF, Hughes TB. Subatmospheric pressure-induced compartment syndrome of the entire upper extremity. A case report. *The Journal Of Bone And Joint Surgery. American Volume*. 2004; **86-A**: 2041-44.

[98] Shah PM, Wapnir I, Babu S, Stahl WM, Clauss RH. Compartment syndrome in combined arterial and venous injuries of the lower extremity. *American Journal Of Surgery*. 1989; **158**: 136-40.

[99] Rocos B, Ward A. Gluteal compartment syndrome with sciatic nerve palsy caused by traumatic rupture of the inferior gluteal artery: a successful surgical treatment. *BMJ case reports*. 2017; **2017**.

[100] Smith A, Chitre V, Deo H. Acute gluteal compartment syndrome: superior gluteal artery rupture following a low energy injury. *BMJ case reports*. 2012; **2012**.

[101] Stringer RW, Mancini M. Blast-Induced Compartment Syndrome. *The Journal of emergency medicine*. 2015; **49**: e91-e92.

[102] Li X, Liang D, Liu X. Compartment syndrome in burn patients. A report of five cases. *Burns: Journal Of The International Society For Burn Injuries*. 2002; **28**: 787-89.

[103] Amundson DE. The spectrum of heat related injury with compartment syndrome. *Military Medicine*. 1989; **154**: 450-52.

[104] Khajavi K, Pavelko T, Mishra AK. Compartment syndrome arising from use of an electronic cooling pad. *The American Journal Of Sports Medicine*. 2004; **32**: 1538-41.

[105] Cancio LC, Jimenez-Reyna JF, Barillo DJ, Walker SC, McManus AT, Vaughan GM. One hundred ninety-five cases of high-voltage electric injury. *The Journal Of Burn Care & Rehabilitation*. 2005; **26**: 331-40.

[106] García Gutiérrez JJ, Meléndez J, Torrero JV, Obregón O, Uceda M, Gabilondo FJ. Lightning injuries in a pregnant woman: a case report and review of the literature. *Burns: Journal Of The International Society For Burn Injuries*. 2005; **31**: 1045-49.

[107] Kikta MJ, Meyer JP, Bishara RA, Goodson SF, Schuler JJ, Flanigan P. Crush syndrome due to limb compression. *Archives Of Surgery (Chicago, Ill.: 1960)*. 1987; **122**: 1078-81.

[108] Iizuka S, Miura N, Fukushima T, Seki T, Sugimoto K, Inokuchi S. Gluteal compartment syndrome due to prolonged immobilization after alcohol intoxication: a case report. *The Tokai Journal Of Experimental And Clinical Medicine*. 2011; **36**: 25-28.

[109] Gerrand CH, Reddy MR, Waldram MA, Simms M. A complication of self-poisoning. *Postgraduate Medical Journal*. 1997; **73**: 113-14.

[110] Cohen RI, Rao R. A 41-year-old man with thigh pain and loss of sensation in the toes. *Chest*. 1997; **111**: 810-12.

[111] Howse AJ, Seddon H. Ischaemic contracture of muscle associated with carbon monoxide and barbiturate poisoning. *British Medical Journal*. 1966; **1**: 192-95.

[112] O'Connor G, McMahon G. Complications of heroin abuse. *European Journal Of Emergency Medicine: Official Journal Of The European Society For Emergency Medicine*. 2008; **15**: 104-06.

[113] Ferrie R, Loveland RC. Bilateral gluteal compartment syndrome after 'ecstasy' hyperpyrexia. *Journal Of The Royal Society Of Medicine*. 2000; **93**: 260-60.

[114] Figueras Coll G, Albortí Fitó G, Iborra Gonzàlez M, Cavanilles Walker JM, Aldea Boniche D, Roca Burniol J. Bilateral compartment syndrome in thighs and legs by methanol intoxication: a case report. *Emergency Medicine Journal: EMJ*. 2008; **25**: 540-41.

[115] Golling M, Fonouni H, Mehrabi A, McArthur N, Huber F-X. Crush syndrome due to drug-induced compartment syndrome: a rare condition not to be overlooked. *Surgery Today*. 2009; **39**: 558-65.

[116] Narayan N, Griffiths M, Patel HDL. Gluteal compartment syndrome with severe rhabdomyolysis. *BMJ case reports*. 2013; **2013**.

[117] Smedick BC, van Wyck D. Lower Extremity Compartment Syndrome From Prolonged Limb Compression and Immobilization During an Airborne Operation. *Journal of special operations medicine : a peer reviewed journal for SOF medical professionals*. 2016; **16**: 5-9.

[118] Abdul-Ghaffar NU, Farghaly MM, Swamy AS. Acute renal failure, compartment syndrome, and systemic capillary leak syndrome complicating carbon monoxide poisoning. *Journal Of Toxicology. Clinical Toxicology*. 1996; **34**: 713-19.

[119] Elliott MJ, Glass KD. Anterior tibial compartment syndrome associated with ergotamine ingestion. *Clinical Orthopaedics And Related Research*. 1976: 44-46.

[120] Lees AJ. Anterior tibial compartment syndrome following prolonged tetany. *Journal Of Neurology, Neurosurgery, And Psychiatry*. 1976; **39**: 406-08.

[121] Lloyd DM, Payne SP, Tomson CR, Barnes MR, Allen MJ. Acute compartment syndrome secondary to theophylline overdose. *Lancet*. 1990; **336**: 312-12.

[122] Vearrier D, Curtis JA. Case files of the medical toxicology fellowship at Drexel University. Rhabdomyolysis and compartment syndrome following acute diphenhydramine overdose. *Journal Of Medical Toxicology: Official Journal Of The American College Of Medical Toxicology*. 2011; **7**: 213-19.

[123] Kasugai D, Tajima K, Jingushi N, Uenishi N, Hirakawa A. Multiple limb compartment syndrome as a manifestation of capillary leak syndrome secondary to metformin and dipeptidyl peptidase IV inhibitor overdose: A case report. *Medicine*. 2020; **99**: e21202.

[124] Farahvash MR, Yegane R-A, Bashashati M, Ahmadi M, Tabrizi N. Surgical approach to hydrocarbon injection in upper extremities: case series. *International Journal Of Surgery (London, England)*. 2009; **7**: 382-86.

[125] Tahir M, Khan MF, Tourbaf K. Impending compartment syndrome and hemothorax after brodifacoum ingestion. *Southern Medical Journal*. 2008; **101**: 1277-77.

[126] Bala I, Pratap M, Nakra D, Ramprabhu T. Prolonged cholinergic crisis and compartment syndrome following subcutaneous injection of an organophosphate compound for suicide attempt. *Journal Of Forensic And Legal Medicine*. 2008; **15**: 256-58.

[127] Bhabra MS, Meshikhes AN, Thomson GJ, Craig P, Parrott NR. Intraarterial temazepam: an important cause of limb ischaemia in intravenous drug abusers. *European Journal Of Vascular Surgery*. 1994; **8**: 240-42.

[128] Witkowski M, Mochmann H-C, Rauch U, Knie W, Landmesser U, Skurk C. Acute Thrombotic Occlusion of the Left Brachial Artery After Intra-Arterial Administration of Amiodarone. *Critical care medicine*. 2016; **44**: e227-e30.

[129] Aynaci O, Onder C, Kalaycioglu A. Anterior tibial compartment syndrome due to the pyomyositis in a patient with rheumatoid arthritis. A case report. *Joint, Bone, Spine: Revue Du Rhumatisme*. 2003; **70**: 77-79.

[130] de Araújo BES, Borchert JM, Manhães PG*, et al.* A rare case of pyomyositis complicated by compartment syndrome caused by ST30-staphylococcal cassette chromosome mec type IV methicillin-resistant Staphylococcus aureus. *The American Journal Of Emergency Medicine*. 2010; **28**: 537.e3-6.

[131] Mulcahey M, Thakur N, Tocci S, Eberson C. Compartment syndrome in a child secondary to acute osteomyelitis of the ulna. *The Pediatric Infectious Disease Journal*. 2009; **28**: 258-59.

[132] Park S, Shatsky JB, Pawel BR, Wells L. Atraumatic compartment syndrome: a manifestation of toxic shock and infectious pyomyositis in a child. A case report. *The Journal Of Bone And Joint Surgery. American Volume*. 2007; **89**: 1337-42.

[133] Dannemann BR, Saffle JR, Stevens GP, Anderson FL, Warden GD. Elevated intramuscular pressure and rhabdomyolysis complicating streptococcal fasciitis. *The Western Journal Of Medicine*. 1984; **140**: 945-48.

[134] Robinson CA, Kellar JZ, Stehr RC. An 84-Year-Old Man with Acute Atraumatic Compartment Syndrome of the Upper Extremity Due to Streptococcus pyogenes Cellulitis. *The American journal of case reports*. 2021; **22**: e929176.

[135] Trellopoulos G, Georgiadis GS, Kapoulas KC, Pitta X, Zervidis I, Lazarides MK. Emergency endovascular treatment of early spontaneous nonaneurysmal popliteal artery rupture in a patient with Salmonella bacteremia. *Journal Of Vascular Surgery: Official Publication, The Society For Vascular Surgery [And] International Society For Cardiovascular Surgery, North American Chapter*. 2010; **52**: 751-57.

[136] Chang-Chien C-H. Bacteraemic necrotizing fasciitis with compartment syndrome caused by non-O1 Vibrio cholerae. *Journal Of Plastic, Reconstructive & Aesthetic Surgery: JPRAS*. 2006; **59**: 1381-84.

[137] Lu Y-H, Lin H-J, Wu K-C. Vibrio vulnificus infection presenting with compartment syndrome of bilateral calves. *The Journal Of Emergency Medicine*. 2008; **35**: 455-56.

[138] Moris V, Chapuis A, Guillier D*, et al.* Necrotizing fasciitis caused by genogroup × Neisseria meningitidis. *QJM : monthly journal of the Association of Physicians*. 2017; **110**: 589-90.

[139] Kim DC, Cresswell A, Mitra A. Compartment syndrome in a patient with X-linked agammaglobulinaemia and ecthyma gangrenosum. Case report. *Scandinavian Journal Of Plastic And Reconstructive Surgery And Hand Surgery / Nordisk Plastikkirurgisk Forening [And] Nordisk Klubb For Handkirurgi*. 2000; **34**: 87-89.

[140] Ternavasio-de la Vega H-G, Marcos-García A, Pisos-Alamo E, Bolaños-Rivero M, Hernández-Cabrera M, Pérez-Arellano J-L. Compartmental syndrome of the upper limb due to Moraxella lacunata infection: a link to patera foot syndrome? *International Journal Of Infectious Diseases: IJID: Official Publication Of The International Society For Infectious Diseases*. 2010; **14 Suppl 3**: e302-e04.

[141] Knox D, Murray G, Millar M*, et al.* Subcutaneous anthrax in three intravenous drug users: a new clinical diagnosis. *The Journal Of Bone And Joint Surgery. British Volume*. 2011; **93**: 414-17.

[142] Gopal V, Burns FE. Cellulitis and compartment syndrome due to Plesiomonas shigelloides: a case report. *Military Medicine*. 1991; **156**: 43-43.

[143] Stull J, Bhat S, Miller AJ, Hoffman R, Wang ML. Treatment of Atypical Compartment Syndrome Due to Proteus Infection. *Orthopedics*. 2017; **40**: e176-e78.

[144] Rewa O, Smith CA. Medical cause of compartment syndrome: a fatal case of Clostridium septicum. *BMJ case reports*. 2012; **2012**.

[145] Silbart S, Oppenheim W. Purpura fulminans. Medical, surgical, and rehabilitative considerations. *Clinical Orthopaedics And Related Research*. 1985: 206-13.

[146] Farnell RD, Davies N, Unwin AJ. Tuberculous compartment syndrome of the forearm. *Orthopedics*. 2001; **24**: 393-94.

[147] Lam R, Lin PH, Alankar S*, et al.* Acute limb ischemia secondary to myositis-induced compartment syndrome in a patient with human immunodeficiency virus infection. *Journal Of Vascular Surgery: Official Publication, The Society For Vascular Surgery [And] International Society For Cardiovascular Surgery, North American Chapter*. 2003; **37**: 1103-05.

[148] Ebbeson RL, De Kock MJ, Penny N, Kollman TR. Rhabdomyolysis, acute renal failure, and compartment syndrome in a child with parainfluenza type 1 infection. *The Pediatric Infectious Disease Journal*. 2009; **28**: 850-52.

[149] Naghibi M, Wijesinghe L, Richardson T. Compartment syndrome: an unusual complication of influenza A infection. *Postgraduate Medicine*. 2008; **120**: 28-31.

[150] Schwerk N, Rokahr C, Hansen G. Cellulitis with compartment syndrome as a complication of varicella zoster infection. *Klinische Pädiatrie*. 2008; **220**: 268-70.

[151] Haq S, Tibby SM, Murdoch IA. Compartment syndrome and Varicella. *The Journal Of Pediatrics*. 1998; **132**: 750-50.

[152] Arslansoyu Çamlar S, Çakir M, Bahat Özdoğan E, Kaya A, Kerimoğlu S, Ökten A. Compartment syndrome, disseminated intravascular coagulation, pneumonia, and acute renal failure due to varicella in a previously healthy child. *Scandinavian journal of infectious diseases*. 2014; **46**: 471-74.

[153] Paletta CE, Lynch R, Knutsen AP. Rhabdomyolysis and lower extremity compartment syndrome due to influenza B virus. *Annals Of Plastic Surgery*. 1993; **30**: 272-73.

[154] Marinella MA. Exertional rhabdomyolysis after recent coxsackie B virus infection. *Southern Medical Journal*. 1998; **91**: 1057-59.

[155] Moghtaderi A, Alavi-Naini R, Azimi H. Compartment syndrome: an unusual course for a rare disease. *The American Journal Of Tropical Medicine And Hygiene*. 2005; **73**: 450-52.

[156] Anam AM, Rabbani R, Shumy F. Spontaneous calf haematoma in severe dengue. *BMJ case reports*. 2018; **2018**.

[157] Case R, Ramaniuk A, Martin P, Simpson PJ, Harden C, Ataya A. Systemic Capillary Leak Syndrome Secondary to Coronavirus Disease 2019. *Chest*. 2020; **158**: e267-e68.

[158] Basaran S, Ozkan C, Coskun-Benlidayi I, Kozanoglu E. Management of a case of human bite complicated by myonecrosis and compartment syndrome. *Journal Of The National Medical Association*. 2009; **101**: 266-69.

[159] Anderson PJ, Zafar I, Nizam M, Berry RB. Compartment syndrome in victims of dog bites. *Injury*. 1997; **28**: 717-17.

[160] Derr C, O'Connor BJ, Macleod SL. Laceration of the popliteal artery and compartment syndrome resulting from stingray envenomation. *The American Journal Of Emergency Medicine*. 2007; **25**: 96-97.

[161] Baird JS. The 'acute compartment syndrome' in snakebite. *Hospital Practice (Office Ed.)*. 1988; **23**: 17-17.

[162] Cohen J, Bush S. Case report: compartment syndrome after a suspected black widow spider bite. *Annals Of Emergency Medicine*. 2005; **45**: 414-16.

[163] Geutjens G. Spontaneous compartment syndrome in a patient with diabetes insipidus. *International Orthopaedics*. 1994; **18**: 53-54.

[164] Thacker AK, Agrawal D, Sarkari NB. Bilateral anterior tibial compartment syndrome in association with hypothyroidism. *Postgraduate Medical Journal*. 1993; **69**: 881-83.

[165] van Veelen NM, Fischli S, Beeres FJP*, et al.* Compartment syndrome of the leg after thyroid hormone withdrawal; two cases and a systematic review of the literature. *BMC endocrine disorders*. 2020; **20**: 80.

[166] Girard-Martel C, Gagnon M. Acute Bilateral Compartment Syndrome Secondary to Polydipsia-Induced Severe Hyponatremia. *Clinical schizophrenia & related psychoses*. 2018; **11**: 197-200.

[167] Luzzi R, Burghardt RD, Herzenberg JE, Zuckerberg AL. Compartment syndrome after hypocalcemic tetany: a case report. *Journal Of Pediatric Orthopedics*. 2008; **28**: 688-90.

[168] Malik SS, Uppal H, Sinha A, Katam K, Srinivasan K. Acute paediatric compartment syndrome of the hand caused by hereditary angiooedema. *Annals Of The Royal College Of Surgeons Of England*. 2011; **93**: e138-e40.

[169] Lak M, Sharifian RA, Karimi K, Mansouritorghabeh H. Acquired hemophilia A: clinical features, surgery and treatment of 34 cases, and experience of using recombinant factor VIIa. *Clinical And Applied Thrombosis/Hemostasis: Official Journal Of The International Academy Of Clinical And Applied Thrombosis/Hemostasis*. 2010; **16**: 294-300.

[170] McQuerry JL, Burnham JM, Ireland ML, Wright RD. Delayed Presentation of Compartment Syndrome of the Thigh in a Previously Undiagnosed Factor VII-Deficient High School Football Athlete: A Case Report. *JBJS case connector*. 2018; **8**: e4.

[171] Alioglu B, Ozsoy MH, Tapci E, Karamercan S, Agras PI, Dallar Y. Successful use of recombinant factor VIIa in a child with Schoenlein-Henoch purpura presenting with compartment syndrome and severe factor XIII deficiency. *Blood coagulation & fibrinolysis : an international journal in haemostasis and thrombosis*. 2013; **24**: 102-05.

[172] Bacal D, Lampman RM, Hogikyan JV, Wolk SW. Compartment syndrome of the arm and disseminated intravascular coagulation. *American Journal Of Orthopedics (Belle Mead, N.J.)*. 2001; **30**: 422-23.

[173] Milanchi S, Magner D. Compartment syndrome of the leg in the coagulopathic, end-stage liver disease patient: Fasciotomy is not the best answer. *International Journal Of Surgery (London, England)*. 2008; **6**: e31-e33.

[174] Dincer HE, Raza T. Compartment syndrome and fatal rhabdomyolysis in sickle cell trait. *WMJ: Official Publication Of The State Medical Society Of Wisconsin*. 2005; **104**: 67-71.

[175] Bedri MI, Khosravi AH, Lifchez SD. Upper extremity compartment syndrome in the setting of deep venous thrombosis and phlegmasia cerulea dolens: case report. *The Journal Of Hand Surgery*. 2009; **34**: 1859-63.

[176] Singh AD, Makkar N, Ray A, Sood R. Phlegmasia cerulea dolens presenting with acute compartment syndrome and pulmonary embolism. *BMJ case reports*. 2018; **2018**.

[177] Triplet JJ, Goss DA, Jr., Taylor B. Spontaneous Compartment Syndrome in a Patient with McArdle Disease: A Case Report and Review of the Literature. *JBJS case connector*. 2017; **7**: e49.

[178] Nishida Y, Tsukushi S, Urakawa H, Arai E, Kozawa E, Ishiguro N. Lower leg compartment syndrome in neurofibromatosis 1 patient with plexiform neurofibrom: a case report of aneurysm rupture. *Annals of vascular surgery*. 2014; **28**: 1035.e5-9.

[179] Joseph VM, Nagy MT, Akhtar S, Ng CY. Sequential spontaneous compartment syndrome in multiple limbs in a young adult with GYG1 gene mutation. *BMJ case reports*. 2020; **13**.

[180] Siegel IM. Compartmental syndrome in Duchenne muscular dystrophy: early evaluation of an epiphenomenon leading to wasting, weakness and contracture. *Medical Hypotheses*. 1992; **38**: 339-45.

[181] Matsushima K, Takara H. Endovascular treatment for a spontaneous rupture of the posterior tibial artery in a patient with Ehlers-Danlos syndrome Type IV: report of a case. *Surgery Today*. 2009; **39**: 523-26.

[182] Pumerantz AW, Stout BJ, Tracy CL. Granulomatosis With Polyangiitis Presenting as Acute Compartment Syndrome. *Journal of clinical rheumatology : practical reports on rheumatic & musculoskeletal diseases*. 2016; **22**: 225-28.

[183] Abidin ASZ, Jalaluddin MY. Left hand compartment syndrome: a rare complication of henoch schonlein purpura. *Journal Of Paediatrics And Child Health*. 2008; **44**: 385-85.

[184] Wirth JJ, Sheka KP, Gheewala A, Rowe NM. Acquired immune deficiency syndrome and systemic lupus erythematosis: potential causes of surgical emergencies of the hand. *Annals Of Plastic Surgery*. 2008; **61**: 35-39.

[185] Pereira de Godoy JM, Braile DM, Torres CAA. Compartment syndrome in patients with Buerger disease and anticardiolipin antibodies. *Southern Medical Journal*. 2008; **101**: 748-49.

[186] Hasaniya N, Katzen JT. Acute compartment syndrome of both lower legs caused by ruptured tibial artery aneurysm in a patient with polyarteritis nodosa: a case report and review of literature. *Journal Of Vascular Surgery: Official Publication, The Society For Vascular Surgery [And] International Society For Cardiovascular Surgery, North American Chapter*. 1993; **18**: 295-98.

[187] Tanagho A, Hatab S, Youssef S, Ansara S. Spontaneous Compartment Syndrome of the Hand in Systemic Sclerosis. *Orthopedics*. 2015; **38**: e849-e51.

[188] Chao YC, Ma CY, Lin LH. Sudden onset of dorsal swelling of hands and feet. *The Netherlands Journal Of Medicine*. 2008; **66**: 307-08.

[189] Murray-Leslie CF, Quinnell RC, Powell RJ, Lowe J. Relapsing eosinophilic myositis causing acute muscle compartment syndrome. *British Journal Of Rheumatology*. 1993; **32**: 436-37.

[190] Alolabi B, Lesieur M, Smilovici B, Koo K, El Bahtimi R, Jenkinson RJ. Forearm compartment syndrome as a result of eosinophilic fasciitis: case report. *The Journal of hand surgery*. 2015; **40**: 707-10.

[191] Hoang C, Porter JM, Ursic CM. Compartment syndrome in multiple uninjured extremities: a case report. *The American Surgeon*. 2002; **68**: 127-29.

[192] Chokshi BV, Lee S, Wolfe SW. Recurrent compartment syndrome of the hand: a case report. *The Journal Of Hand Surgery*. 1998; **23**: 66-69.

[193] Lamou H, Grassmann J-P, Betsch M*, et al.* Systemic capillary leak syndrome associated with a rare abdominal and four-limb compartment syndrome: a case report. *Journal of medical case reports*. 2014; **8**: 196.

[194] Parker PJ, Evans MJ, Annan IH. Catastrophic familial rhabdomyolysis: compartment syndrome with muscle fiber regeneration. *Journal Of Pediatric Orthopaedics. Part B*. 1999; **8**: 64-66.

[195] Bayar A, Keser S, Hosnuter M, Tanriverdi HA, Ege A. Lower limb compartment syndrome after an uncomplicated labor. *Orthopedics*. 2007; **30**: 972-73.

[196] Jyothi NK, Cox C. Compartment syndrome following postpartum haemorrhage. *BJOG: An International Journal Of Obstetrics And Gynaecology*. 2000; **107**: 430-32.

[197] Coulton S, Bourne S, Catliffe S, Brooks R, Jollow D. Acute compartment syndrome of the lower limb following childbirth: a case report. *Journal of medical case reports*. 2020; **14**: 140.

[198] Martin B, Treharne L. Neonatal compartment syndrome. *Annals of the Royal College of Surgeons of England*. 2016; **98**: e111-e13.

[199] Tontchev G, Silverberg NB, Shlasko E, Henry C, Roberts JL, Roth MZ. Techniques for toddlers: linear band incision for harlequin ichthyosis with associated compartment syndrome. *Pediatric dermatology*. 2014; **31**: 625-29.

[200] Chandrasekaran N, Yudin MH, Berger H. Uterine Diverticulum With Fetal Leg Entrapment: A Case Report. *Journal of obstetrics and gynaecology Canada : JOGC = Journal d'obstetrique et gynecologie du Canada : JOGC*. 2017; **39**: 894-96.

[201] Bekmez S, Beken S, Mermerkaya MU, Ozkan M, Okumus N. Acute forearm compartment syndrome in a newborn caused by reperfusion after spontaneous axillary artery thrombosis. *Journal of pediatric orthopedics. Part B*. 2015; **24**: 552-55.

[202] Dotan M, Nahum E, Weigl D, Bilavsky E. Compartment syndrome because of acute hemorrhagic edema of infancy: a case report and literature review. *Journal of pediatric orthopedics. Part B*. 2014; **23**: 419-21.

[203] Badawy SM, Gust MJ, Liem RI, Ball MK, Gosain AK, Sharathkumar AA. Neonatal Compartment Syndrome Associated With Disseminated Intravascular Coagulation. *Annals of plastic surgery*. 2016; **76**: 256-58.

[204] Christiansen SD, Desai NS, Pulito AR, Slack MR. Ischemic extremities due to compartment syndromes in a septic neonate. *Journal Of Pediatric Surgery*. 1983; **18**: 641-43.

[205] Downey-Carmona FJ, González-Herranz P, De La Fuente-González C, Castro M. Acute compartment syndrome of the foot caused by a hemangioma. *The Journal Of Foot And Ankle Surgery: Official Publication Of The American College Of Foot And Ankle Surgeons*. 2006; **45**: 52-55.

[206] Joseph FR, Posner MA, Terzakis JA. Compartment syndrome caused by a traumatized vascular hamartoma. *The Journal Of Hand Surgery*. 1984; **9**: 904-07.

[207] Chim CS, Choy C, Liang R. Primary anaplastic large cell lymphoma of skeletal muscle presenting with compartment syndrome. *Leukemia & Lymphoma*. 1999; **33**: 601-05.

[208] Simmons DJ, Wharton SM, Waters R. Compartment syndrome complicating metastatic malignant melanoma. *British Journal Of Plastic Surgery*. 2000; **53**: 255-57.

[209] Lee D-K, Jeong W-K, Lee D-H, Lee S-H. Multiple compartment syndrome in a pediatric patient with CML. *Journal Of Pediatric Orthopedics*. 2011; **31**: 889-92.

[210] Southworth SR, O'Malley NP, Ebraheim NA, Zeff L, Cummings V. Compartment syndrome as a presentation of non-Hodgkin's lymphoma. *Journal Of Orthopaedic Trauma*. 1990; **4**: 470-73.

[211] Trumble T. Forearm compartment syndrome secondary to leukemic infiltrates. *The Journal Of Hand Surgery*. 1987; **12**: 563-65.

[212] Veeragandham RS, Paz IB, Nadeemanee A. Compartment syndrome of the leg secondary to leukemic infiltration: a case report and review of the literature. *Journal Of Surgical Oncology*. 1994; **55**: 198-200.

[213] Schneider I, Müller T, Stoltenburg G, Holzhausen H-J, Mawrin C, Hanisch F. Pseudo-Popeye syndrome: extramedullary plasmacytoma manifesting in skeletal muscle. *Neurology*. 2014; **82**: 544-45.

[214] Van Oost J, Feyen J, Opheide J. Compartment syndrome associated with an osteocartilaginous exostosis. *Acta Orthopaedica Belgica*. 1996; **62**: 233-35.

[215] Ward WG, Eckardt JJ. Ganglion cyst of the proximal tibiofibular joint causing anterior compartment syndrome. A case report and anatomical study. *The Journal Of Bone And Joint Surgery. American Volume*. 1994; **76**: 1561-64.

[216] Garayoa SA, Romero-Muñoz LM, Pons-Villanueva J. Acute compartment syndrome of the forearm caused by calcific tendinitis of the distal biceps. *Musculoskeletal Surgery*. 2010; **94**: 137-39.

[217] Dunlop D, Parker PJ, Keating JF. Ruptured Baker's cyst causing posterior compartment syndrome. *Injury*. 1997; **28**: 561-62.

[218] Batt M, Sosa M, Bouillanne P-J, Thevenin B, Haudebourg P, Hassen-Khodja R. Acute compartment syndrome: an unusual complication of a previously bypassed popliteal aneurysm--case report and literature review. *Journal Of Vascular Surgery: Official Publication, The Society For Vascular Surgery [And] International Society For Cardiovascular Surgery, North American Chapter*. 2006; **43**: 1049-52.

[219] Hammoudeh M, Siam AR, Khanjar I. Anterior dissection of popliteal cyst causing anterior compartment syndrome. *The Journal Of Rheumatology*. 1995; **22**: 1377-79.

[220] Karkos CD, Hughes R, Prasad V, D'Souza SP. Thigh compartment syndrome as a result of a false aneurysm of the profunda femoris artery complicating fixation of an intertrochanteric fracture. *The Journal Of Trauma*. 1999; **47**: 393-95.

[221] Matsagas MI, Mitsis M, Rigopoulos C*, et al.* A large radial artery false aneurysm after repeated arterial punctures, causing compartment syndrome of the forearm. *Intensive Care Medicine*. 2003; **29**: 1032-32.

[222] McCaffer C, Lip G, Bachoo P, Rajagopalan S. Infective endocarditis causing ulnar mycotic aneurysm and compartment syndrome. *Postgraduate medical journal*. 2012; **88**: 426-27.

[223] Das SS, Behera S, Das G, Patro BP. Compartment syndrome as a late presentation of brachial artery pseudoaneurysm following shaft of humerus fracture. *BMJ case reports*. 2019; **12**.

[224] Hanypsiak B, Bergfeld JA, Miniaci A, Joyce MJ. Recurrent compartment syndrome after fracture of a tibiofibular synostosis in a National Football League player. *The American Journal Of Sports Medicine*. 2007; **35**: 127-30.

[225] Sgouros S, Ali MS. An unusual cause of carpal tunnel syndrome. Case report. *Scandinavian Journal Of Plastic And Reconstructive Surgery And Hand Surgery / Nordisk Plastikkirurgisk Forening [And] Nordisk Klubb For Handkirurgi*. 1992; **26**: 335-37.

[226] Bird CB, McCoy JW, Jr. Weight-lifting as a cause of compartment syndrome in the forearm. A case report. *The Journal Of Bone And Joint Surgery. American Volume*. 1983; **65**: 406-06.

[227] Kuklo TR, Tis JE, Moores LK, Schaefer RA. Fatal rhabdomyolysis with bilateral gluteal, thigh, and leg compartment syndrome after the Army Physical Fitness Test. A case report. *The American Journal Of Sports Medicine*. 2000; **28**: 112-16.

[228] Bertoldo U, Nicodemo A, Pallavicini J, Massè A. Acute bilateral compartment syndrome of the thigh induced by spinning training. *Injury*. 2003; **34**: 791-92.

[229] Adesina O-OO, Beall DP, Madden GW, Hurst RB. Leg pain in a young soccer player: the diagnosis of acute exertional compartment syndrome. *The Journal Of The Oklahoma State Medical Association*. 2009; **102**: 82-84.

[230] Middleton DK, Johnson JE, Davies JF. Exertional compartment syndrome of bilateral feet: a case report. *Foot & Ankle International / American Orthopaedic Foot And Ankle Society [And] Swiss Foot And Ankle Society*. 1995; **16**: 95-96.

[231] Hill CE, Modi CS, Baraza N, Mosleh-Shirazi MS, Dhukaram V. Spontaneous compartment syndrome of the foot. *The Journal Of Bone And Joint Surgery. British Volume*. 2011; **93**: 1282-84.

[232] Dietrich D, Paley KJ, Ebraheim NA. Spontaneous tibial compartment syndrome: case report. *The Journal Of Trauma*. 1994; **37**: 138-39.

[233] O'Leary EJP, Bulstrode NW, Gschwind C. Acute bilateral forearm compartment syndrome of unknown aetiology. *Hand Surgery: An International Journal Devoted To Hand And Upper Limb Surgery And Related Research: Journal Of The Asia-Pacific Federation Of Societies For Surgery Of The Hand*. 2006; **11**: 147-49.

[234] Kowalewski K, Mayo A, Journeaux S. How a footballer got hurt without getting hit: isolated peroneal compartment syndrome of a non-traumatic cause. *Annals Of The Royal College Of Surgeons Of England*. 2007; **89**: W1-W2.

[235] Neth MR. Acute hand pain resulting in spontaneous thenar compartment syndrome. *The American journal of emergency medicine*. 2019; **37**: 561.e3-61.e4.

[236] Large TM, Frick SL. Compartment syndrome of the leg after treatment of a femoral fracture with an early sitting spica cast. A report of two cases. *The Journal Of Bone And Joint Surgery. American Volume*. 2003; **85-A**: 2207-10.

[237] Danner R, Partanen K, Partanen J, Kettunen K. Iatrogenic compartment syndrome, A follow-up of four cases caused by elastic bandage. *Clinical Neurology And Neurosurgery*. 1989; **91**: 37-43.

[238] Weitz EM, Carson G. The anterior tibial compartment syndrome in a twenty month old infant. (A complication of the use of a bow leg brace). *Bulletin Of The Hospital For Joint Diseases*. 1969; **30**: 16-20.

[239] Dunwoody JM, Reichert CC, Brown KL. Compartment syndrome associated with bupivacaine and fentanyl epidural analgesia in pediatric orthopaedics. *Journal Of Pediatric Orthopedics*. 1997; **17**: 285-88.

[240] Greene TL, Louis DS. Compartment syndrome of the arm--a complication of the pneumatic tourniquet. A case report. *The Journal Of Bone And Joint Surgery. American Volume*. 1983; **65**: 270-73.

[241] Hinderland MD, Ng A, Paden MH, Stone PA. Lateral leg compartment syndrome caused by ill-fitting compression stocking placed for deep vein thrombosis prophylaxis during surgery: a case report. *The Journal Of Foot And Ankle Surgery: Official Publication Of The American College Of Foot And Ankle Surgeons*. 2011; **50**: 616-19.

[242] Vogel LC, Lubicky JP. Lower extremity compartment syndrome in an adolescent with spinal cord injury. *The Journal Of Spinal Cord Medicine*. 2001; **24**: 278-83.

[243] Lachmann EA, Rook JL, Tunkel R, Nagler W. Complications associated with intermittent pneumatic compression. *Archives Of Physical Medicine And Rehabilitation*. 1992; **73**: 482-85.

[244] Aprahamian C, Gessert G, Bandyk DF, Sell L, Stiehl J, Olson DW. MAST-associated compartment syndrome (MACS): a review. *The Journal Of Trauma*. 1989; **29**: 549-55.

[245] Handler EG. Superficial compartment syndrome of the foot after infiltration of intravenous fluid. *Archives Of Physical Medicine And Rehabilitation*. 1990; **71**: 58-59.

[246] Chen T-K, Yang C-Y, Chen S-J. Calcinosis cutis complicated by compartment syndrome following extravasation of calcium gluconate in a neonate: a case report. *Pediatrics And Neonatology*. 2010; **51**: 238-41.

[247] Santivasi WL, Kulkarni S, Patton ML*, et al.* Infiltration of sodium valproate with compartment syndrome and bullous reaction: case report and literature review. *Burns: Journal Of The International Society For Burn Injuries*. 2011; **37**: e59-e62.

[248] Edwards JJ, Samuels D, Fu ES. Forearm compartment syndrome from intravenous mannitol extravasation during general anesthesia. *Anesthesia And Analgesia*. 2003; **96**: 245.

[249] Alexander CM, Ramseyer M, Beatty JS. Missed Extravasation Injury from Peripheral Infusion of Norepinephrine Resulting in Forearm Compartment Syndrome and Amputation. *The American surgeon*. 2016; **82**: e162-e63.

[250] Varacallo M, Shirey L, Kavuri V, Harding S. Acute compartment syndrome of the hand secondary to propofol extravasation. *Journal of clinical anesthesia*. 2018; **47**: 1-2.

[251] Chhabra P, Gupta N, Kaushik A. Compartment syndrome as a spectrum of purple glove syndrome following intravenous phenytoin administration in a young male: a case report and review of literature. *Neurology India*. 2013; **61**: 419-20.

[252] Atanda A, Jr., Statter MB. Compartment syndrome of the leg after intraosseous infusion: guidelines for prevention, early detection, and treatment. *American Journal Of Orthopedics (Belle Mead, N.J.)*. 2008; **37**: E198-E200.

[253] Ananthanarayan C, Castro C, McKee N, Sakotic G. Compartment syndrome following intravenous regional anesthesia. *Canadian Journal Of Anaesthesia = Journal Canadien D'anesthésie*. 2000; **47**: 1094-98.

[254] Younge D. Haematoma block for fractures of the wrist: a cause of compartment syndrome. *Journal Of Hand Surgery (Edinburgh, Scotland)*. 1989; **14**: 194-95.

[255] Egert R. Diagnostic peripheral nerve block resulting in compartment syndrome. *American Journal Of Physical Medicine & Rehabilitation / Association Of Academic Physiatrists*. 1988; **67**: 230-30.

[256] Kontrobarsky Y, Love J. Gluteal compartment syndrome following epidural analgesic infusion with motor blockage. *Anaesthesia And Intensive Care*. 1997; **25**: 696-98.

[257] Green G. A fatal case of malignant hyperthermia complicated by generalized compartment syndrome and rhabdomyolysis. *Acta Anaesthesiologica Scandinavica*. 2003; **47**: 619-21.

[258] Smith DC, Mitchell DA, Peterson GW, Will AD, Mera SS, Smith LL. Medial brachial fascial compartment syndrome: anatomic basis of neuropathy after transaxillary arteriography. *Radiology*. 1989; **173**: 149-54.

[259] Tsao BE, Wilbourn AJ. The medial brachial fascial compartment syndrome following axillary arteriography. *Neurology*. 2003; **61**: 1037-41.

[260] Ghasseimi A, Salman M, Tiwari A. Compartment syndrome of the forearm following brachial artery puncture and subsequent anticoagulation. *Journal Of Interventional Cardiology*. 2002; **15**: 435-35.

[261] Horlocker TT, Bishop AT. Compartment syndrome of the forearm and hand after brachial artery cannulation. *Anesthesia And Analgesia*. 1995; **81**: 1092-94.

[262] Shabat S, Carmel A, Cohen Y*, et al.* Iatrogenic forearm compartment syndrome in a cardiac intensive care unit induced by brachial artery puncture and acute anticoagulation. *Journal Of Interventional Cardiology*. 2002; **15**: 107-09.

[263] Araki T, Itaya H, Yamamoto M. Acute compartment syndrome of the forearm that occurred after transradial intervention and was not caused by bleeding or hematoma formation. *Catheterization And Cardiovascular Interventions: Official Journal Of The Society For Cardiac Angiography & Interventions*. 2010; **75**: 362-65.

[264] Bertrand OF. Acute forearm muscle swelling post transradial catheterization and compartment syndrome: prevention is better than treatment! *Catheterization And Cardiovascular Interventions: Official Journal Of The Society For Cardiac Angiography & Interventions*. 2010; **75**: 366-68.

[265] Halpern AA, Mochizuki R, Long CE, 3rd. Compartment syndrome of the forearm following radial-artery puncture in a patient treated with anticoagulants. *The Journal Of Bone And Joint Surgery. American Volume*. 1978; **60**: 1136-37.

[266] Lin Y-J, Chu C-C, Tsai C-W. Acute compartment syndrome after transradial coronary angioplasty. *International Journal Of Cardiology*. 2004; **97**: 311-11.

[267] Qvist J, Peterfreund RA, Perlmutter GS. Transient compartment syndrome of the forearm after attempted radial artery cannulation. *Anesthesia And Analgesia*. 1996; **83**: 183-85.

[268] Safran MR, Bernstein A, Lesavoy MA. Forearm compartment syndrome following brachial arterial puncture in uremia. *Annals Of Plastic Surgery*. 1994; **32**: 535-38.

[269] Tizón-Marcos H, Barbeau GR. Incidence of compartment syndrome of the arm in a large series of transradial approach for coronary procedures. *Journal Of Interventional Cardiology*. 2008; **21**: 380-84.

[270] Alameddine AK. Lower limb ischemia with compartment syndrome related to femoral artery cannulas. *The Annals Of Thoracic Surgery*. 1997; **64**: 884-85.

[271] Selby IR, Darowski MJ. Compartment syndrome in a child occurring after femoral artery cannulation. *Paediatric Anaesthesia*. 1995; **5**: 393-95.

[272] Rosengart R, Nelson RJ, Emmanoulides GC. Anterior tibial compartment syndrome in a child: an unusual complication of cardiac catheterization. *Pediatrics*. 1976; **58**: 456-58.

[273] Gates JD, Bichell DP, Rizzo RJ, Couper GS, Donaldson MC. Thigh ischemia complicating femoral vessel cannulation for cardiopulmonary bypass. *The Annals Of Thoracic Surgery*. 1996; **61**: 730-33.

[274] Vaz AJ. Compartmental syndrome following subclavian vein hemodialysis. *Clinical And Experimental Dialysis And Apheresis*. 1982; **6**: 15-24.

[275] Alsafadi H. Medical image. Ecchymotic wrist drop. *The New Zealand Medical Journal*. 2009; **122**: 61-62.

[276] Asplund MW. Acute thigh compartment syndrome post femoral vein catheterization: a case report. *WMJ: Official Publication Of The State Medical Society Of Wisconsin*. 2008; **107**: 244-46.

[277] Garrett RC, Kerstein MD. Compartment syndrome in the newborn. *Southern Medical Journal*. 1987; **80**: 533-34.

[278] Wood KE, Reedy JS, Pozniak MA, Coursin DB. Phlegmasia cerulea dolens with compartment syndrome: a complication of femoral vein catheterization. *Critical Care Medicine*. 2000; **28**: 1626-30.

[279] Reddy SP, Matta S, Handa A. Forearm compartment syndrome following puncture of haemodialysis access fistula. *European Journal Of Vascular And Endovascular Surgery: The Official Journal Of The European Society For Vascular Surgery*. 2002; **23**: 458-59.

[280] Mesfin A, Lum YW, Nayfeh T, Mears SC. Compartment syndrome in patients with massive venous thrombosis after inferior vena cava filter placement. *Orthopedics*. 2011; **34**: 229-29.

[281] Shokoohi H, Smith J, Holmes A, Abell B. Bilateral compartment syndrome as a result of inferior vena cava filter thrombosis. *Annals Of Emergency Medicine*. 2008; **52**: 104-07.

[282] Su WT, Stone DH, Lamparello PJ, Rockman CB. Gluteal compartment syndrome following elective unilateral internal iliac artery embolization before endovascular abdominal aortic aneurysm repair. *Journal Of Vascular Surgery: Official Publication, The Society For Vascular Surgery [And] International Society For Cardiovascular Surgery, North American Chapter*. 2004; **39**: 672-75.

[283] Sanchez de Toledo J, Chrysostomou C, Wearden PD. Acute compartment syndrome in a patient on extracorporeal support: utility of near-infrared spectroscopy. *Journal Of Cardiothoracic And Vascular Anesthesia*. 2011; **25**: 836-37.

[284] Wall CJ, Santamaria J. Extracorporeal membrane oxygenation: an unusual cause of acute limb compartment syndrome. *Anaesthesia And Intensive Care*. 2010; **38**: 560-62.

[285] Velez CA, Kahn J. Compartment syndrome from balloon pump. *Catheterization And Cardiovascular Interventions: Official Journal Of The Society For Cardiac Angiography & Interventions*. 2000; **51**: 217-19.

[286] Alms M. Compartmental syndrome as a complication of the Hauser Procedure. *The Journal Of Bone And Joint Surgery. American Volume*. 1980; **62**: 313-13.

[287] Wall JJ. Compartment syndrome as a complication of the Hauser procedure. *The Journal Of Bone And Joint Surgery. American Volume*. 1979; **61**: 185-91.

[288] Wiggins HE. The anterior tibial compartmental syndrome. A complication of the Hauser procedure. *Clinical Orthopaedics And Related Research*. 1975: 90-94.

[289] Schweitzer DL, Aguam AS, Wilder JR. Complications encountered during arterial embolectomy with the Fogarty balloon catheter. *Vascular Surgery*. 1976; **10**: 144-56.

[290] Jacob ZC, Tito MF, Dagum AB. MR imaging-related electrical thermal injury complicated by acute carpal tunnel and compartment syndrome: case report. *Radiology*. 2010; **254**: 846-50.

[291] Apostle KL, Heran MKS, Tredwell SJ. Acute compartment syndrome after sclerotherapy for a low-flow vascular malformation. *Canadian Journal Of Surgery. Journal Canadien De Chirurgie*. 2008; **51**: E50-E51.

[292] Cho YP, Kim E, Choi S-J*, et al.* Compartment syndrome after compression sclerotherapy. *Annals Of Vascular Surgery*. 2005; **19**: 428-30.

[293] Rheingold LM, Fater MC, Courtiss EH. Compartment syndrome of the upper extremity following cutaneous laser surgery. *Plastic And Reconstructive Surgery*. 1997; **99**: 1418-20.

[294] Brewer MB, Folstein MK, Kerns M, Jesse E. Compartment syndrome of the thigh as a complication of electromyography. *The American Surgeon*. 2012; **78**: 72-73.

[295] Farrell CM, Rubin DI, Haidukewych GJ. Acute compartment syndrome of the leg following diagnostic electromyography. *Muscle & Nerve*. 2003; **27**: 374-77.

[296] Vaienti L, Vourtsis S, Urzola V. Compartment syndrome of the forearm following an electromyographic assessment. *Journal Of Hand Surgery (Edinburgh, Scotland)*. 2005; **30**: 656-57.

[297] Roth JS, Newman EC. Gluteal compartment syndrome and sciatica after bone marrow biopsy: a case report and review of the literature. *The American Surgeon*. 2002; **68**: 791-94.

[298] Faulhaber J, Ehmke H, Koenen W, Weiss B, Goerdt S, Schneider SW. Compartment syndrome of the lower leg after punch biopsy. *Dermatologic Surgery: Official Publication For American Society For Dermatologic Surgery [Et Al.]*. 2012; **38**: 277-78.

[299] Ramachandran M, Lau K, Jones DHA. Rotational osteotomies for congenital radioulnar synostosis. *The Journal Of Bone And Joint Surgery. British Volume*. 2005; **87**: 1406-10.

[300] Moholkar K, Smyth H. Acute compartment syndrome of the forearm in association with ulnar shortening osteotomy: a case report. *The Journal Of Hand Surgery*. 2000; **25**: 358-59.

[301] Baeten Y, De Smet L, Fabry G. Acute anterior forearm compartment syndrome following wrist arthrodesis. *Acta Orthopaedica Belgica*. 1999; **65**: 239-41.

[302] Jones CD, Addison PR, Lam WL, Davidson DM. Compartment Syndrome of the Forearm Following Dermofasciectomy-A Rare and Devastating Complication. *The journal of hand surgery Asian-Pacific volume*. 2019; **24**: 491-93.

[303] Ploumis A, Casnellie M, Graber JN, Dykes DC. Acute tibial compartment syndrome following spine surgery. *Orthopedics*. 2010; **33**: 447-47.

[304] Magaji SA, Debnath UK, Mehdian HS. Compartment syndrome of leg following total lumbar disc replacement via anterior retroperitoneal approach: a rare complication of anterior spinal surgery. *Spine*. 2010; **35**: E74-E76.

[305] Rahóty P, Szendröi M. Tumor surgery of the pelvic region. *Acta Chirurgica Hungarica*. 1997; **36**: 284-85.

[306] Kumar V, Saeed K, Panagopoulos A, Parker PJ. Gluteal compartment syndrome following joint arthroplasty under epidural anaesthesia: a report of 4 cases. *Journal Of Orthopaedic Surgery (Hong Kong)*. 2007; **15**: 113-17.

[307] Marsh A, Johnstone D, Stott D. Compartment syndrome as a complication of positioning for revision hip arthroplasty--a case report. *Acta Orthopaedica Scandinavica*. 2000; **71**: 527-29.

[308] Bezwada HP, Nazarian DG, Booth RE, Jr. Compartment syndrome following total knee arthroplasty: a case report. *American Journal Of Orthopedics (Belle Mead, N.J.)*. 2005; **34**: 386-88.

[309] Kort NP, van Raay JJAM, van Horn JR. Compartment syndrome and popliteal vascular injury complicating unicompartmental knee arthroplasty. *The Journal Of Arthroplasty*. 2007; **22**: 472-76.

[310] Ciftci S, Gulec A, Mercan N, Yildrim A. A new complication of femur head core decompression surgery: compartment syndrome. *JPMA. The Journal of the Pakistan Medical Association*. 2020; **70**: 1642-44.

[311] Tommaso OD, Nisticò A, Vitullo A. Unusual complication during knee arthroscopy. *Anaesthesia*. 2004; **59**: 305-06.

[312] Krysa J, Lofthouse R, Kavanagh G. Gluteal compartment syndrome following posterior cruciate ligament repair. *Injury*. 2002; **33**: 835-38.

[313] Price C, Ribeiro J, Kinnebrew T. Compartment syndromes associated with postoperative epidural analgesia. A case report. *The Journal Of Bone And Joint Surgery. American Volume*. 1996; **78**: 597-99.

[314] Boonstra RH, Haverkamp D, Campo MM, van der Vis HM. Acute compartment syndrome of the thigh following total knee arthroplasty. *The Knee*. 2012; **19**: 151-53.

[315] Miller WR. Volkmann's contracture of the lower extremity; a complication of patellar tendon transplantation. *United States Armed Forces Medical Journal*. 1956; **7**: 1517-20.

[316] Tavares JO. Acute compartment syndrome in osteochondromatosis. *Orthopedics*. 2004; **27**: 775-76.

[317] Almdahl SM, Due J, Jr., Samdal FA. Compartment syndrome with muscle necrosis following repair of hernia of tibialis anterior. Case report. *Acta Chirurgica Scandinavica*. 1987; **153**: 695-95.

[318] Reed J, Hiemstra LA. Anterior compartment syndrome following an Achilles tendon repair: an unusual complication. *Clinical Journal Of Sport Medicine: Official Journal Of The Canadian Academy Of Sport Medicine*. 2004; **14**: 237-41.

[319] Taras JS, Fitzpatrick MJ. Compartment syndrome of the leg after plantaris tendon harvest: a case report. *The Journal Of Hand Surgery*. 2001; **26**: 1135-37.

[320] Imade S, Takao M, Miyamoto W, Nishi H, Uchio Y. Leg anterior compartment syndrome following ankle arthroscopy after Maisonneuve fracture. *Arthroscopy: The Journal Of Arthroscopic & Related Surgery: Official Publication Of The Arthroscopy Association Of North America And The International Arthroscopy Association*. 2009; **25**: 215-18.

[321] Lauber S, Schulte TL, Götze C, Steinbeck J, Bottner F. Acute compartment syndrome following intramedullary pulse lavage and débridement for osteomyelitis of the tibia. *Archives Of Orthopaedic And Trauma Surgery*. 2005; **125**: 564-66.

[322] Seiler JG, 3rd, Valadie AL, 3rd, Drvaric DM, Frederick RW, Whitesides TE, Jr. Perioperative compartment syndrome. A report of four cases. *The Journal Of Bone And Joint Surgery. American Volume*. 1996; **78**: 600-02.

[323] Naito M, Ogata K. Acute volar compartment syndrome during skeletal traction in distal radius fracture. A case report. *Clinical Orthopaedics And Related Research*. 1989: 234-37.

[324] Divani KG, O'Brien A, Molloy S, Trivedi J, Cowan J, Gibson A. A multicentre retrospective review of muscle necrosis of the leg following spinal surgery with motor evoked potential monitoring: a cause for concern? *European spine journal : official publication of the European Spine Society, the European Spinal Deformity Society, and the European Section of the Cervical Spine Research Society*. 2016; **25**: 801-06.

[325] Patil SD, Patil VD, Abane S, Luthra R, Ranaware A. Acute Compartment Syndrome of the Foot due to Infection After Local Hydrocortisone Injection: A Case Report. *The Journal of foot and ankle surgery : official publication of the American College of Foot and Ankle Surgeons*. 2015; **54**: 692-96.

[326] Al-Sarraf N, Al-Shammari F, Vislocky I, Malek L. Lower limb compartment syndrome following coronary artery bypass surgery: a rare entity. *General Thoracic And Cardiovascular Surgery*. 2010; **58**: 131-33.

[327] Palmer BV, Mercer JL. Anterior tibial compartment syndrome following femoral artery perfusion. *Thorax*. 1973; **28**: 492-94.

[328] Rosenfield AL, Bartal E. Bilateral spontaneous lateral compartment syndrome in the legs of a patient who received a kidney and heart transplant. A case report. *The Journal Of Bone And Joint Surgery. American Volume*. 1992; **74**: 775-76.

[329] Oates M, Saxena P, Cosic F, Yadav S. Bilateral gluteal compartment syndrome after thoracic surgery: an unusual complication. *ANZ journal of surgery*. 2016; **86**: 952-53.

[330] Nishino M, Okano M, Kawada J, Kim Y, Yamada M, Tsujinaka T. Well-leg compartment syndrome after laparoscopic low anterior resection for lower rectal cancer in the lithotomy position: A case report. *Asian journal of endoscopic surgery*. 2018; **11**: 53-55.

[331] Passari G, Lentini S, Benedetto F, La Spada M, Spinelli F. Cross-femoral venous by-pass (Palma's procedure) to relieve venous hypertension due to retroperitoneal leiomyosarcoma: a case report. *Acta Chirurgica Belgica*. 2010; **110**: 383-86.

[332] Hauser J, Lehnhardt M, Steinau H-U, Homann H-H. Trocar injury of the retroperitoneal vessels followed by life-threatening postischemic compartment syndrome of both lower extremities. *Surgical Laparoscopy, Endoscopy & Percutaneous Techniques*. 2008; **18**: 222-24.

[333] Honda T, Tokushige M, Uda S, Egawa H, Suginami H. A case of laparoscopic complication: injury of the left common iliac vessels and subsequent acute compartment syndrome of the left leg. *Journal Of Obstetrics And Gynaecology (Tokyo, Japan)*. 1995; **21**: 273-75.

[334] Nakayama R, Katagiri A, Ando T, Mizusawa T. Common iliac artery thrombosis as a possible complication of the pluck technique in nephroureterectomy. *BMJ case reports*. 2018; **2018**.

[335] Ferreira TA, Pensado A, Dominguez L, Aymerich H, Molins N. Compartment syndrome with severe rhabdomyolysis in the postoperative period following major vascular surgery. *Anaesthesia*. 1996; **51**: 692-94.

[336] Osamura N, Takahashi K, Endo M, Kurumaya H, Shima I. Lumbar paraspinal myonecrosis after abdominal vascular surgery: a case report. *Spine*. 2000; **25**: 1852-54.

[337] O'Leary R, McAree B, Bell MDD, Troxler M, Jackson P. Gluteal compartment syndrome presenting with features of iatrogenic epidural haematoma. *British Journal Of Anaesthesia*. 2010; **104**: 510-11.

[338] Chew MH, Xu GG, Ho PW, Lee CW. Gluteal compartment syndrome following abdominal aortic aneurysm repair: a case report. *Annals Of Vascular Surgery*. 2009; **23**: 535.e15-20.

[339] Ferreira J, Galle C, Aminian A*, et al.* Lumbar paraspinal rhabdomyolysis and compartment syndrome after abdominal aortic aneurysm repair. *Journal Of Vascular Surgery: Official Publication, The Society For Vascular Surgery [And] International Society For Cardiovascular Surgery, North American Chapter*. 2003; **37**: 198-201.

[340] Ishibashi H, Ohta T, Hosaka M, Sugimoto I, Kawanishi J, Yamada T. Gluteal compartment syndrome after abdominal aortic aneurysm repair. *VASA. Zeitschrift Für Gefässkrankheiten. Journal For Vascular Diseases*. 2004; **33**: 89-91.

[341] Pua BB, Muhs BE, Cayne NS, Dobryansky M, Jacobowitz GR. Bilateral gluteal compartment syndrome after elective unilateral hypogastric artery ligation and revascularization of the contralateral hypogastric artery during open abdominal aortic aneurysm repair. *Journal Of Vascular Surgery: Official Publication, The Society For Vascular Surgery [And] International Society For Cardiovascular Surgery, North American Chapter*. 2005; **41**: 337-39.

[342] Saleem SM, van Doorn CA. A swollen shoulder after repair of acute dissection of the aorta: an unusual presentation of a compartment syndrome. *The Journal Of Thoracic And Cardiovascular Surgery*. 2001; **122**: 627-28.

[343] Best IM, Bumpers HL. Thigh compartment syndrome after acute ischemia. *The American Surgeon*. 2002; **68**: 996-98.

[344] Maharaj D, Naraynsingh V, Kuruvilla T. Compartment syndrome due to reperfusion injury. *The West Indian Medical Journal*. 1996; **45**: 125-26.

[345] Persson NH, Bergqvist D, Takolander R. Lower-limb oedema after thromboembolectomy for acute arterial occlusion. *Acta Chirurgica Scandinavica*. 1990; **156**: 603-08.

[346] Quinn RH, Ruby ST. Compartment syndrome after elective revascularization for chronic ischemia. A case report and review of the literature. *Archives Of Surgery (Chicago, Ill.: 1960)*. 1992; **127**: 865-66.

[347] Whatling PJ, Galland RB. Isolated compartment syndrome of the hand after brachial embolectomy. *European Journal Of Vascular And Endovascular Surgery: The Official Journal Of The European Society For Vascular Surgery*. 1999; **17**: 446-47.

[348] Clayton DG. Two cases of compartment syndrome in the intensive care unit. *Intensive Care Medicine*. 1986; **12**: 432-34.

[349] Pereira de Godoy JM, Meziara JC, Braile DM. Compartment syndrome in subcutaneous and skin tissue of a dialysis patient operated for creation of an AV fistula. *International Urology And Nephrology*. 2005; **37**: 437-38.

[350] Wang K-L, Li S-Y, Chuang C-L, Chen T-W, Chen J-Y. Subfascial hematoma progressed to arm compartment syndrome due to a nontransposed brachiobasilic fistula. *American Journal Of Kidney Diseases: The Official Journal Of The National Kidney Foundation*. 2006; **48**: 990-92.

[351] Beadnell SW, Saunderson JR, Sorenson DC. Compartment syndrome following oral and maxillofacial surgery. *Journal Of Oral And Maxillofacial Surgery: Official Journal Of The American Association Of Oral And Maxillofacial Surgeons*. 1988; **46**: 232-34.

[352] Teeples TJ, Rallis DJ, Rieck KL, Viozzi CF. Lower extremity compartment syndrome associated with hypotensive general anesthesia for orthognathic surgery: a case report and review of the disease. *Journal Of Oral And Maxillofacial Surgery: Official Journal Of The American Association Of Oral And Maxillofacial Surgeons*. 2010; **68**: 1166-70.

[353] Strickland SM, Westrich GH. Spontaneous compartment syndrome occurring postoperatively in 2 oral surgery patients. *Journal Of Oral And Maxillofacial Surgery: Official Journal Of The American Association Of Oral And Maxillofacial Surgeons*. 2000; **58**: 814-16.

[354] Lecky B. Acute bilateral anterior tibial compartment syndrome after Caesarian section in a diabetic. *Journal Of Neurology, Neurosurgery, And Psychiatry*. 1980; **43**: 88-90.

[355] Radosa JC, Radosa MP, Sütterlin M. Acute compartment syndrome in obstetric care. *Acta Obstetricia Et Gynecologica Scandinavica*. 2011; **90**: 677-77.

[356] Oláh KS. Adductor compartment syndrome: An unusual complication of the trans-obturator tape procedure. *Journal Of Obstetrics And Gynaecology: The Journal Of The Institute Of Obstetrics And Gynaecology*. 2008; **28**: 363-64.

[357] Addison PD, Lannon D, Neligan PC. Compartment syndrome after closure of the anterolateral thigh flap donor site: a report of two cases. *Annals Of Plastic Surgery*. 2008; **60**: 635-38.

[358] Saleem M, Hashim F, Babu Manohar M. Compartment syndrome in a free fibula osteocutaneous flap donor site. *British Journal Of Plastic Surgery*. 1998; **51**: 405-07.

[359] Rajoo R, Mennen U, Stevanovic M. Compartment syndrome in transferred muscle: an unusual complication. *Journal Of Hand Surgery (Edinburgh, Scotland)*. 1991; **16**: 75-77.

[360] Hallock GG. Myonecrosis as a sequela of calf implants. *Annals Of Plastic Surgery*. 1993; **30**: 456-58.

[361] Stotts AK, Carroll KL, Schafer PG, Santora SD, Branigan TD. Medial compartment syndrome of the foot: an unusual complication of spine surgery. *Spine*. 2003; **28**: E118-E20.

[362] Godeiro-Júnior CO, Oliveira ASB, Felício AC, Barros N, Gabbai AA. Peroneal nerve palsy due to compartment syndrome after facial plastic surgery. *Arquivos De Neuro-Psiquiatria*. 2007; **65**: 826-29.

[363] Karmaniolou I, Staikou C. Compartment syndrome as a complication of the lithotomy position. *The West Indian Medical Journal*. 2010; **59**: 698-701.

[364] Turnbull D, Mills GH. Compartment syndrome associated with the Lloyd Davies position. Three case reports and review of the literature. *Anaesthesia*. 2001; **56**: 980-87.

[365] Noordin S, Allana S, Wajid. Well leg compartment syndrome: the debit side of hemilithotomy position. *Journal Of Ayub Medical College, Abbottabad: JAMC*. 2009; **21**: 166-68.

[366] Rudolph T, Løkebø JE, Andreassen L. Bilateral gluteal compartment syndrome and severe rhabdomyolysis after lumbar spine surgery. *European Spine Journal: Official Publication Of The European Spine Society, The European Spinal Deformity Society, And The European Section Of The Cervical Spine Research Society*. 2011; **20 Suppl 2**: S180-S82.

[367] Cascio BM, Buchowski JM, Frassica FJ. Well-limb compartment syndrome after prolonged lateral decubitus positioning. A report of two cases. *The Journal Of Bone And Joint Surgery. American Volume*. 2004; **86-A**: 2038-40.

[368] Ahmad FU, Madhavan K, Trombly R, Levi AD. Anterior thigh compartment syndrome and local myonecrosis after posterior spine surgery on a Jackson table. *World neurosurgery*. 2012; **78**: 553.e5-8.

[369] O'Connor D, Breslin D, Barry M. Well-leg compartment syndrome following supine position surgery. *Anaesthesia And Intensive Care*. 2010; **38**: 595-95.

[370] Clark JM, Friedell ML, Gupta BR, Davenport WC, Amponsah K. Perioperative compartment syndrome of the hand. *The American Surgeon*. 2011; **77**: 116-17.

[371] Galyon SW, Richards KA, Pettus JA, Bodin SG. Three-limb compartment syndrome and rhabdomyolysis after robotic cystoprostatectomy. *Journal Of Clinical Anesthesia*. 2011; **23**: 75-78.

[372] Zimmerman DC, Kapoor T, Elfond M, Scott P. Spontaneous compartment syndrome of the upper arm in a patient receiving anticoagulation therapy. *The Journal of emergency medicine*. 2013; **44**: e53-e56.

[373] Calabro LJ, Dick CG, Lutz MJ. Acute compartment syndrome of the thigh following minor trauma in a patient on dual anti-platelet therapy. *Emergency Medicine Australasia: EMA*. 2011; **23**: 95-97.

[374] Byrne AM, Kearns SR, Kelly EP. Posterior compartment syndrome associated with clopidogrel therapy following trivial trauma. *Emergency Medicine Journal: EMJ*. 2006; **23**: 697-98.

[375] Limberg RM, Dougherty C, Mallon WK. Enoxaparin-induced bleeding resulting in compartment syndrome of the thigh: a case report. *The Journal Of Emergency Medicine*. 2011; **41**: e1-e4.

[376] Porras MC, Bonilla BC, Gómez EP, Ruiz MG. Dalteparin-induced extremity hematoma complicated by probable compartment syndrome. *The Annals Of Pharmacotherapy*. 2001; **35**: 643-45.

[377] Beall S, Garner J, Oxley D. Anterolateral compartment syndrome related to drug-induced bleeding. A case report. *The American Journal Of Sports Medicine*. 1983; **11**: 454-55.

[378] Hannon MG, Lamont JG. Compartment syndrome due to massive leg hematoma after primary total hip arthroplasty: a previously unreported complication of fondaparinux. *The Journal of arthroplasty*. 2012; **27**: 1414.e9-14.e11.

[379] Crick KA, Crick JC, Pulley MT. Hemorrhagic upper extremity complications from tissue plasminogen activator. *Journal Of Surgical Orthopaedic Advances*. 2007; **16**: 27-30.

[380] Möller MG, Lewis JM, Dessureault S, Zager JS. Toxicities associated with hyperthermic isolated limb perfusion and isolated limb infusion in the treatment of melanoma and sarcoma. *International Journal Of Hyperthermia: The Official Journal Of European Society For Hyperthermic Oncology, North American Hyperthermia Group*. 2008; **24**: 275-89.

[381] Thomas GP, Kadam S, Mohammed M, Andrews BTE. Compartment syndrome as a rare complication of iloprost infusion for peripheral vascular disease. *Annals Of Vascular Surgery*. 2011; **25**: 555.e11-2.

[382] De Sousa R, Dang A, Rataboli PV. Nicolau syndrome following intramuscular benzathine penicillin. *Journal Of Postgraduate Medicine*. 2008; **54**: 332-34.

[383] Knapke DM, Truumees E. Posterior arm and deltoid compartment syndrome after vitamin B12 injection. *Orthopedics*. 2004; **27**: 520-21.

[384] Varoga D, Drescher W, Lippross S, Pufe T, Schütz R. Nonsteroidal anti-inflammatory drug (NSAID)-related spontaneous compartment syndrome resulting from severe platelet dysfunction. *The Journal Of Trauma*. 2009; **66**: 1251-52.

[385] Chagnac A, Wisnovitz M, Zevin D, Korzets A, Mittelman M, Levi J. Cyclosporin-associated rhabdomyolysis and anterior compartment syndrome in a renal transplant recipient. *Clinical Nephrology*. 1993; **39**: 351-52.

[386] Chow LT, Chow WH. Acute compartment syndrome: an unusual presentation of gemfibrozil induced myositis. *The Medical Journal Of Australia*. 1993; **158**: 48-49.

[387] Walker JL, Smith GH, Gaston MS, Robinson CM. Spontaneous compartment syndrome in association with simvastatin-induced myositis. *Emergency Medicine Journal: EMJ*. 2008; **25**: 305-06.

[388] Higgs D, da Assunção R. Atraumatic forearm compartment syndrome: alert patients taking neuroleptics are at risk. *Injury*. 2004; **35**: 1200-01.

[389] Clarissa Samara V, Warner J. Rare case of severe serotonin syndrome leading to bilateral compartment syndrome. *BMJ case reports*. 2017; **2017**.

[390] Oh LS, Lewis PB, Prasarn ML, Lorich DG, Helfet DL. Painless, atraumatic, isolated lateral compartment syndrome of the leg: an unusual triad of atypical findings. *American Journal Of Orthopedics (Belle Mead, N.J.)*. 2010; **39**: 35-39.

[391] Graham B, Loomer RL. Anterior compartment syndrome in a patient with fracture of the tibial plateau treated by continuous passive motion and anticoagulants. Report of a case. *Clinical Orthopaedics And Related Research*. 1985: 197-99.

[392] Bergqvist D. Vascular injuries caused by acupuncture. *European Journal Of Vascular And Endovascular Surgery: The Official Journal Of The European Society For Vascular Surgery*. 2008; **36**: 160-63.
